# Variations in *O*-Glycosylation Patterns Influence Viral Pathogenicity, Infectivity, and Transmissibility in SARS-CoV-2 Variants

**DOI:** 10.3390/biom13101467

**Published:** 2023-09-29

**Authors:** Sherifdeen Onigbinde, Cristian D. Gutierrez Reyes, Mojibola Fowowe, Oluwatosin Daramola, Mojgan Atashi, Andrew I. Bennett, Yehia Mechref

**Affiliations:** Department of Chemistry and Biochemistry, Texas Tech University, Lubbock, TX 79409, USA; sonigbin@ttu.edu (S.O.); cristian.d.gutierrez-reyes@ttu.edu (C.D.G.R.); mfowowe@ttu.edu (M.F.); odaramol@ttu.edu (O.D.); mojgan.atashi@ttu.edu (M.A.); andy.bennett@ttu.edu (A.I.B.)

**Keywords:** SARS-CoV-2, mutations, *O*-glycosylation, pathogenicity, transmissibility, LC-MS/MS

## Abstract

The highly glycosylated S protein plays a vital role in host cell invasion, making it the principal target for vaccine development. Differences in mutations observed on the spike (S) protein of SARS-CoV-2 variants may result in distinct glycosylation patterns, thus influencing immunological evasion, infectivity, and transmissibility. The glycans can mask key epitopes on the S1 protein and alter its structural conformation, allowing the virus to escape the immune system. Therefore, we comprehensively characterize *O*-glycosylation in eleven variants of SARS-CoV-2 S1 subunits to understand the differences observed in the biology of the variants. In-depth characterization was performed with a double digestion strategy and an efficient LC-MS/MS approach. We observed that *O*-glycosylation is highly conserved across all variants in the region between the NTD and RBD, whereas other domains and regions exhibit variation in *O*-glycosylation. Notably, omicron has the highest number of *O*-glycosylation sites on the S1 subunit. Also, omicron has the highest level of sialylation in the RBD and RBM functional motifs. Our findings may shed light on how differences in *O*-glycosylation impact viral pathogenicity in variants of SARS-CoV-2 and facilitate the development of a robust vaccine with high protective efficacy against the variants of concern.

## 1. Introduction

The COVID-19 pandemic, which is caused by the SARS-CoV-2 virus, a member of the β-coronaviruses, caught the world by storm, claiming millions of lives worldwide and altering the pace and fashion of global systems [1]. As of 7 September 2023, 770,563,467 cases of COVID-19 and 6,957,216 deaths were confirmed and reported to the World Health Organization [2]. After the SARS-CoV-2 outbreak in late 2019, there was an 11-month stretch of relative evolutionary stability of the virus. However, since late 2020, SARS-CoV-2 has undergone several mutations at a fast pace to produce variants of concern (VOCs) and variants of interest (VOIs) with observed differences in their transmissibility and infectivity [3,4]. The VOCs include the alpha, beta, delta, gamma, and omicron variants while the others are referred to as VOIs. One of the mutations that emerged and increased in frequency was an amino acid substitution, D614G, within the C-terminal domain (CTD) of the viral spike protein. This mutation was shown by some studies to confer a reasonable benefit for transmissibility [5] and infectivity [6,7]. Another notable missense mutation that emerged within the receptor-binding motif (RBM) of the S1 protein of some variants is N501Y. This mutation has been reported to be more infectious than D614G [8,9] and exists in the current variant of concern, omicron.

The viral transmembrane spike (S) glycoprotein, a trimeric class 1 fusion protein made up of two functional subunits, S1 and S2, is the most essential component of SARS-CoV-2 pathogenesis. It contains the receptor-binding domain (RBD) in the S1 subunit that interacts with the angiotensin-converting enzyme 2 (ACE2) receptor of host cells to facilitate viral entry into the cell [10,11,12,13,14], and the S2 subunit that enables the membrane fusion of viral and host cells [15,16]. The cleavage site at the S1/S2 boundary, which oversees activating β-coronaviruses S proteins, is another important region on the S1 subunit [17,18]. The furin cleavage process plays a crucial role in increasing the receptor binding and fusion activity of the spike protein, contributing significantly to SARS-CoV-2 transmission [19,20,21,22]. Of particular interest is the heavy glycosylation of the S1 subunit that serves as a form of “glycan shield” [23,24]. This glycan shield significantly influences the overall pathogenesis of the SARS-CoV-2 virus and protects it from the activity of neutralizing antibodies produced by the host immune response. As a result, the S1 subunit is very important and is the primary target for generating vaccines and neutralizing antibodies in clinical trials [25,26]. 

Comprehensive research has been conducted to decipher the pattern of *N*-glycosylation on S proteins in SARS-CoV-2 and other members of the β-coronaviruses, such as MERS-CoV and SARS-CoV [16,27,28,29,30,31]. Although SARS-CoV-2 was reported to exhibit a lesser glycan shield with more exposed areas due to fewer *N*-glycosylation sites compared to other β-coronaviruses [27,32], it is still more pathogenic and transmissible [33,34]. *N*-glycosylation plays a crucial role in various aspects of SARS-CoV-2 infection, including viral binding and entrance [35,36], as well as immune recognition and response [37,38]. Thus, an in-depth study of the specific glycosylation patterns of recombinant viral S proteins may provide essential insights into viral biology and direct vaccine design tactics [39,40].

Like viral *N*-glycosylation, *O*-glycosylation also influences viral entrance, spread, and glycan shielding and is linked with infectivity and evolutionary adaptation of the virus [41,42,43,44]. Several groups have characterized *O*-glycosylation on the S protein of the wild-type SARS-CoV-2 virus, utilizing different expression systems [29,31,45,46,47,48,49]. A recent study investigated *O*-glycosylation changes in SARS-CoV-2 VOCs, but their findings are limited to the prevalent *O*-glycosylation site T323 [30]. Recent reports have shown that *O*-glycosylation can influence furin-mediated spike cleavage impacting infectivity and transmissibility in SARS-CoV-2 [44,50]. Increased proteolytic processing of spike S protein in some VOCs has been associated with P681 mutation to His or Arg amino acid residues [51,52,53]. It has been demonstrated that these mutations decrease *O*-glycosylation on Serine (S) or Threonine (T) on a peptide stretch proximal to the furin cleavage site, thereby increasing proteolytic cleavage of S protein into S1 and S2 subunits [44,50]. Consequently, this increase in proteolytic cleavage increases the infectivity of the VOCs. Unlike *N*-glycosylation sites, *O*-glycosylation sites on SARS-CoV-2 S proteins do not have a conserved sequon, a common sugar core, or a high occupancy of glycans; rather, they are occupied with a variety of *O*-linked glycans with low occupancy [27,45,54]. As a result, the comprehensive study of viral *O*-glycosylation is greatly impeded. This necessitates a dire need for greater efforts toward *O*-glycosylation studies of the SARS-CoV-2 S protein.

Since adaptive immunity and infectivity are influenced by viral glycosylation [32,55], mutational alterations in the amino acid sequence of the SARS-CoV-2 S protein variants may produce new sites of glycosylation that could contribute to the specific viral biology of each variant. For example, we identified *O*-glycosylation on mutation points R190S and F490S observed in the Gamma and Lambda variants, respectively. There is limited knowledge about the comprehensive *O*-glycosylation studies of the different SARS-CoV-2 S1 protein variants that exist within the global population. Therefore, we performed a qualitative and quantitative in-depth analysis to determine the *O*-glycosylation patterns of eleven variants of the SARS-CoV-2 S1 protein using high-resolution liquid chromatography–mass spectrometry (LC-MS/MS). To address an accurate and efficient characterization of the S1 protein’s *O*-glycosylation, we employed two strategies. Initially, double enzymatic digestion using trypsin and immunomodulating metalloprotease (IMPa) was performed, followed by a paired MS/MS dissociation strategy using HCD and EThcD. IMPa is an *O*-glycoprotease that cleaves *N*-terminally to S or T linked by a mucin-type *O*-glycan, and sialylation on the *O*-linked glycan does not affect its digestion efficiency [56,57]. This approach allowed a confident assignment of isobaric *O*-glycopeptide forms by differentiating the S or T *O*-glycosylation position. In addition, the analysis revealed an extensive heterogeneity in the *O*-glycosylation pattern in the different SARS-CoV2 S1 protein variants. Thus, by describing the comprehensive *O*-glycosylation patterns of the SARS-CoV-2 S1 protein variants, our findings may reveal how variation in *O*-glycosylation impacts infection and transmission of SARS-CoV-2 variants and serve as a foundation for the development of vaccines with a broad spectrum of functionality.

## 2. Materials and Methods

### 2.1. Chemicals and Reagents

Trypsin/Lys-C mix mass spectrometry grade was purchased from Promega (Madison, WI, USA). *O*-glycoprotease (IMPa) was obtained from New England Biolabs (Ipswich, MA). HPL C grade acetonitrile (MeCN) and water were purchased from Fisher Scientific (Fair Lawn, NJ, USA). Formic acid (FA), ammonium bicarbonate (ABC), dithiothreitol (DTT), and iodoacetamide (IAA) were purchased from Sigma Aldrich (St. Louis, MO, USA). SARS CoV-2 (2019-nCoV) Spike S1 protein variants were purchased from Sino Biologicals (US Inc., Wayne, PA, USA) (Table 1).

### 2.2. Tryptic and IMPa Digestion

The tryptic digestion was performed according to the method previously described by Gutierrez Reyes et al. [58]. Briefly, 10 µL of glycoproteins were reconstituted with 40 µL of 50 mM ABC buffer and denatured for 15 min at 90 °C in a water bath. The denatured glycoprotein was reduced by the addition of 1.25 µL of 200 mM DTT and incubated at 60 °C for 45 min. Following that, the glycoprotein was alkylated by adding 5.0 µL of 200 mM IAA and incubated at 37 °C for 45 min. To quench the excess IAA, another 1.25 µL of 200 mM DTT was added and incubated at 37 °C for 30 min. Thereafter, trypsin/Lys-C enzyme was introduced in a 1:25 enzyme-to-protein ratio and incubated at 37 °C for 20 h. After incubation, the digestion was quenched by heating at 90 °C for 15 min and dried under vacuum in a SpeedVac concentrator (Labconco CentriVap). In a second enzymatic digestion, the tryptic-digested samples were resuspended in 20 mM Tris-HCl solution (pH = 8.0) to a total reaction volume of 50 µL. The IMPa enzyme was added at a 1:10 enzyme-to-protein ratio and incubated at 37 °C for 20 h. The samples were finally dried in a SpeedVac concentrator and resuspended in mobile phase A (MPA) containing 98% water, 2% ACN, and 0.1% formic acid. All samples were prepared in biological triplicates to demonstrate reproducibility.

### 2.3. LC-MS/MS Analysis 

The reconstituted tryptic/IMPa digests were resuspended to a final concentration of 500 ng/µL. Two microliters of the reconstituted sample were injected into an Acclaim PepMap 100 C18 trap (75 mm × 2 cm, 3 mm particle size, Thermo Scientific, Pittsburg, PA, USA) for online purification. Thereafter, the sample was separated on a reversed-phase C18 Acclaim PepMap 100 Å capillary column (150 mm × 75 μm id, Thermo Scientific, Pittsburg, PA, USA) with a temperature of 40 °C and a flow rate of 0.35 μL/min. The chromatographic gradient started at 2% of mobile phase B (MPB) for 5 min and gradually increased to 30% over 35 min, and 70% over 32 min. Then, it was ramped up to 90% of MPB in 1 min and kept constant for 7 min to wash the system. Finally, it was decreased to 2% MPB in 1 min and kept constant for 9 min to equilibrate the column for the next injection. The LC-MS/MS analysis of the *O*-glycopeptide samples was performed with an UltiMate 3000 nano-LC system (Dionex, Sunnyvale, CA, USA) coupled to an Orbitrap Fusion Lumos Tribrid Mass Spectrometer (Thermo Scientific, San Jose, CA, USA). The MPA consisted of 98% water and 2% ACN containing 0.1% FA, and the MPB consisted of 100% ACN containing 0.1% FA. After the LC separation, the *O*-glycopeptide samples were introduced into the mass spectrometer using a nano ESI source in positive ion mode, 2 kV, and a transfer tube temperature of 275 °C. The full MS spectra were acquired in an Orbitrap mass analyzer with a mass range of 500 to 1800 *m*/*z*, with a resolving power of 120 K and a mass accuracy of 5 ppm. The RF lens was set at 60% and the maximum injection time was 50 ms. The dynamic exclusion parameters were set as follows: repeat count 1; exclusion duration of 60 s; mass tolerance of 10 ppm; and an intensity threshold of 5.0 × 10^4^. The MS/MS orbitrap scans were generated in data-dependent acquisition (DDA) mode with a duty cycle of 3 s and a selection of the 20 most intense ions from the full MS scan. For the *O*-glycopeptide dissociation, an initial high-energy collision dissociation followed by an electron-transfer/higher-energy collision dissociation (HCD-EThcD) strategy was applied. For the initial HCD, a normalized collision energy (NCE) of 35% and 10 ms of activation time were utilized. The isolation window was 2 *m*/*z* with a resolution of 30 k, a fixed scan range of 120 to 4000 *m*/*z*, and a maximum injection time of 60 ms. Then, the most intense ions were acquired and sent to a second MS/MS scan using EThcD with an isolation window of 1.6 *m*/*z*. A second MS/MS scan was generated using an EThcD dissociation. The Quadrupole was utilized for ion isolation with a window of 1.6 *m*/*z*. The ETD reaction time was 50 ms with a reagent target of 2.0 × 10^5^ and a maximum ETD reagent injection time of 200 ms. The supplemental HCD was set at 25%. The generated fragment ions were analyzed in the Orbitrap with a resolution of 30 K, a fixed scan range of 120 to 4000 *m*/*z*, and a maximum injection time of 200 ms. 

### 2.4. Data Processing

The raw data files were first processed in Byonic software (version 4.1.10, Protein Metrics, Inc., Cupertino, CA, USA) for *O*-glycopeptide identification, with a mass tolerance of 10 ppm for precursors and 20 ppm for fragment ions. Carbamidomethyl (C) was set as a fixed modification, while oxidation (M), acetyl (protein *N*-terminal), and deamidation (N) were variable modifications. In the digestion parameters, cleavage sites RK and ST were specified with cleavage sides at the C-terminal and *N*-terminal for Trypsin and IMPa, respectively. MetaMorpheus software (version 0.0.320) was also utilized for the identification of *O*-glycopeptides according to the *O*-pair search method described by Lu et al. [59]. Identification was followed by a manual check of the peak retention time, monoisotopic mass, and mass spectra using XCalibur software^®^ (Version 2.2, Thermo Scientific) to validate the identified *O*-glycopeptides and eliminate false positive ones. The fragment ions were theoretically validated using Glycoworkbench (Version 2.0) [60]. The areas under the peaks were quantified to represent the abundance of each identified *O*-glycopeptide. Relative quantification of the *O*-glycopeptides was accomplished in Microsoft Excel (version 2308) by normalizing the area by the total abundance. The bar plots and heatmaps were generated using GraphPad Prism 9, and Biorender software was used to generate the workflow schematic.

The findings of this study indicate that a significant majority, specifically over 80%, of the *O*-glycopeptides that were unambiguously identified do not possess the consensus sequon required for *N*-glycosylation, which suggests a minimal influence of *N*-linked glycans on the identification of *O*-linked glycans. Nevertheless, in the case of *O*-glycosylation occurring on the serine (S) or threonine (T) amino acid residues neighboring asparagine within the consensus sequon, only glycopeptides classified as level 1 by MetaMorpheus were deemed unequivocal, as the distinctive fragments associated with these glycopeptides accurately pinpoint the glycosylation site on the S/T amino acid residue. This type of glycosylation was manually investigated and validated.

## 3. Results and Discussion

### 3.1. Comprehensive Analysis of Site-Specific O-Glycosylation

**Figure 1:**
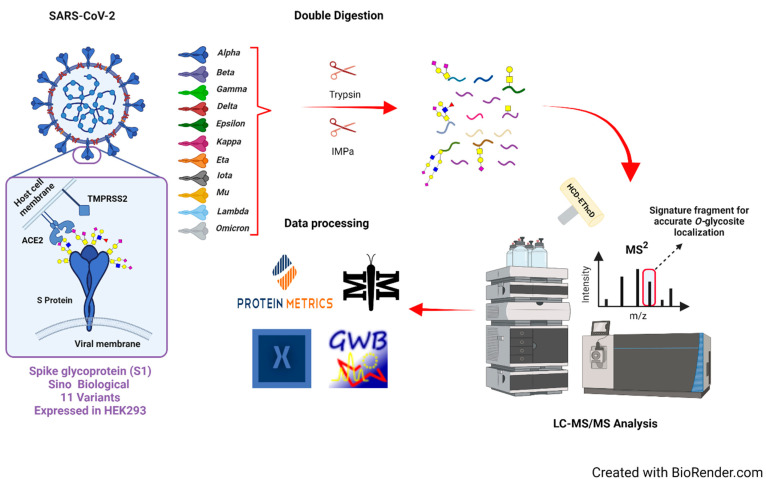
Schematics of the *O*-glycoproteomic workflow employed in this work. Variants of SARS-CoV-2 S1 proteins recombinantly expressed in HEK293 were digested using trypsin followed by IMPa. The glycopeptide digests were analyzed by LC-MS/MS. Glycopeptides were identified using Byonic (version 4.1.10) and MetaMorpheus software (version 1.0.1). The *O*- glycan symbols used in this work: 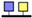, HexNAc (N); 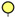, Hex (H); 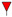, Fucose (F); and 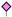, NeuAc (A).

As shown in Figure 1, we devised an approach to conduct a thorough investigation of the *O*-glycosylation on the S1 proteins from eleven SARS-CoV-2 variants. The investigated variants were kappa, alpha, epsilon, gamma, delta, beta, iota, eta, lambda, mu, and omicron. To rule out any potential bias introduced by the expression of the spike proteins, all the variants utilized for this study were expressed in HEK293 cells. The investigated S1 proteins have different amino acid sequences as a result of mutations including substitution, insertion, or deletion at different regions or domains in the spike proteins of the different SARS-CoV-2 variants, as shown in Table 1. The validation of the mutant S1 glycoproteins was completed through LC-MS proteomics analysis. The protein coverage was calculated utilizing Proteome Discoverer 2.5 software (Thermo Scientific). The results demonstrate the association of the analyzed S1 proteins with the SARS-CoV-2 variants (Supplementary Figure S1).

The *O*-glycoproteomics experiment was initiated with the tryptic digestion of the S1 protein variants. Then, the tryptic digests were subjected to a second digestion using the *O*-glycoprotease IMPa enzyme, which cleaves *N*-terminally to Ser/Thr amino acid residues that are *O*-glycosylated. A high-resolution LC-MS/MS approach was utilized to examine the *O*-glycopeptides. The mutations described by the vendor (Table 1) were used to construct the S1 protein sequences for the different variants. This information was uploaded into Byonic^®^ (version 4.1.10) and MetaMorpheus^®^ software (version 1.0.1) to complete the *O*-glycopeptide identification, followed by manual validation of the detected precursors. The MetaMorpheus^®^ software (version 1.0.1) was utilized according to the *O*-pair search approach described by Lu et al. [59]. The Level 1 searches were reported as spectral evidence that confidently assigns glycans to the glycosites.

**Figure 2:**
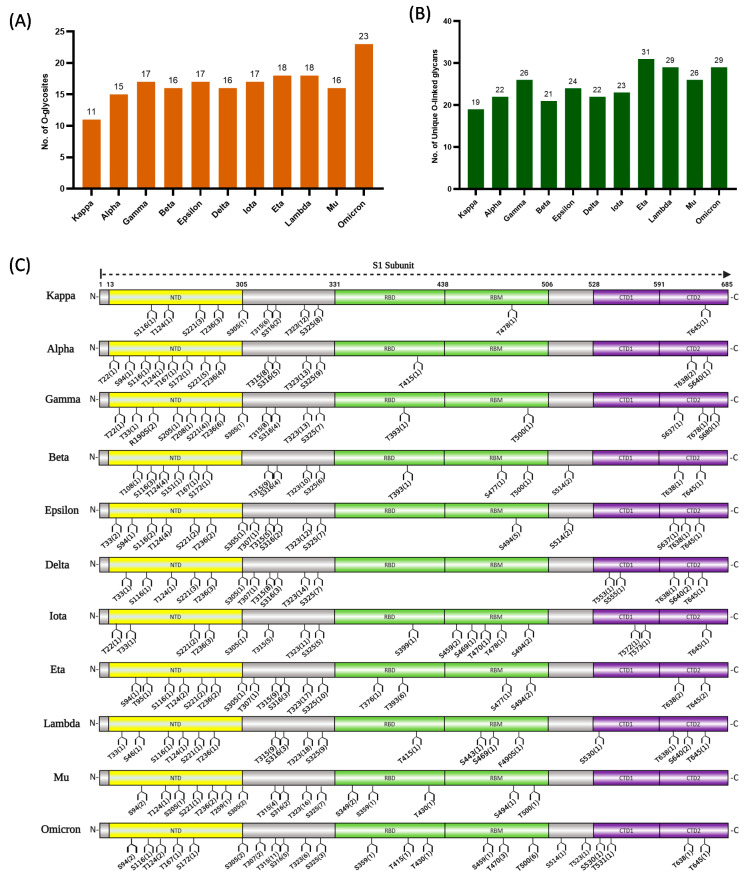
Site-specific localization of *O*-glycoforms in SARS-CoV-2 variants S1 proteins. The bar graphs represent the number of (**A**) unambiguous *O*-glycosylation sites and (**B**) unique *O*-linked glycans observed in each SARS-CoV-2 variant. The error bar represents the standard deviation (n = 3). (**C**) Schematic representation of the 11 SARS-CoV-2 variants S1 glycoprotein showing the position of *O*-linked glycosylation. The domains and regions of the S1 glycoproteins are illustrated as NTD (yellow), the region between NTD and RBD 305–331 (grey), RBD (green), RBM (green), and CTD 1/2 (purple). The identified *O*-glycopeptides are shown in detail in Supplementary Tables S1 and S2 for Byonic and MetaMorpheus, respectively.

The comparisons of the *O*-glycopeptides identified in the S1 subunits of the SARS-CoV-2 variants showed extensive heterogeneity. Figure 2A shows the number of glycosylation sites observed in the analyzed variants, while Figure 2B shows the number of unique *O*-glycans attached to the unambiguously identified *O*-glycosites. The different strains show the distribution of the *O*-glycosylation sites across different regions and domains of the S1 subunit of the SARS-CoV-2 S protein (Figure 2C). These include the *N*-terminal domain (NTD), receptor binding domain (RBD), receptor binding motif (RBM), C-terminal domain (CTD), and the region between the NTD and RBD designated as amino acid residues 305–331. The designation of the different domains and regions of the S1 protein was determined according to the X-ray crystallography structure of SARS-CoV-2 RBD complexed with ACE2 [61]. According to these results, the omicron, eta, and lambda variants were more densely *O*-glycosylated with 23, 18, and 18 sites, respectively. The variants with fewer glycosylation sites were alpha and kappa with 15 and 11 sites, respectively. Importantly, all data were generated in biological triplicates, and identical results were obtained from the replicates, demonstrating the reproducibility of our analytic method. Previously, 11 *O*-glycosylation sites have been identified on the S1 subunit of the wild-type (WT) SARS-CoV-2 spike protein while 6 sites were found on the S2 subunit [46]. Liang and co-workers unambiguously identified 16 *O*-glycosylation sites in the S1 subunit of the WT spike protein and 14 in its D614G mutant expressed in HEK 293 cells [47]. This observation implies that mutations have the potential to influence *O*-glycosylation in spike proteins. Additionally, the authors reported the identification of 6 *O*-glycosylation sites on the S1 subunit of the WT SARS-CoV-2 that was expressed in insects. This significant change in the number of *O*-glycosylation sites may be attributable to the different expression systems utilized. Wang et al. conducted a comparative analysis of *N*- and *O*-glycosylation patterns in HEK293 and Baculovirus insect cells [48]. Their findings revealed distinct variations in the site-specific glycosylation of spike proteins when expressed in these two different systems. Additionally, it was revealed that there is a notable disparity in the sialylation distribution of *N*-glycans between HEK293 cells and Baculovirus insect cells. The authors suggested that the expression systems may exhibit preferences for *O*-glycosylation enzymes, particularly GalNAcTs, which could account for the observed variances in *O*-glycosylation. Another study has documented notable disparities in the *O*-glycosylation patterns of the receptor-binding domain (RBD) when expressed as a monomer, as opposed to its localization on the dimeric ectodomain of insect cells [45]. However, they reported similarities in *O*-glycosylation patterns between insect and HEK293 ectodomains. In the work of Zhang and colleagues, 3 *O*-glycosites were confidently assigned in the full-length S protein expressed in the insect cell while 11 sites were assigned in only the S1 subunit expressed in the human cell [49]. In addition, the reported sialylation in the S1 subunit expressed in human cells increased compared to insect cells. The utilization of recombinant viral proteins as a foundation for the development of non-mRNA vaccines [62,63] necessitates a comprehensive understanding of the glycosylation characteristics shown by these recombinant viral proteins across various cellular systems. It has been demonstrated that HEK293 cells adhere to the general mammalian glycosylation pathway; thus, it was explored in this study [64]. Figure 2B shows the number of *O*-linked glycans derived from the studied variants. Similar to the work of Dong et al., we have identified some unique *O*-linked glycans in SARS-CoV-2 S1 proteins including H2N3F2, H1N2F2, and H2N3F3 [47]. We have also shown multiple sialylated *O*-glycans such as H1N1A3 and H1N2A3 linked to the S1 protein in some variants. In addition, we identified large *O*-glycans, including H4N6F2 in alpha and lambda; H5N5F3 in lambda; and H4N4F3A1 in lambda, beta, eta, and mu. Overall, our findings showed extensive differences in the *O*-glycosylation of the S1 proteins of SARS-CoV-2 variants.

Important differences in the S1 protein domains and regions were also observed. For example, the RBM was *O*-glycosylated in all the variants except alpha and delta (Figure 2C). In all the SARS-CoV-2 variants characterized in this approach, the region between NTD and RBD with the designated amino acid residues 305–331 was found to be extensively *O*-glycosylated and conserved among all variants. Thr 323 in this region has been reported to be extensively *O*-glycosylated in other studies as well [29,30,31,65]. While the delta variant exhibits no unambiguous *O*-glycosylation in the RBD and RBM functional motifs, either or both the domain and the motif were mostly *O*-glycosylated in the other variants. In the omicron variant, Roberts et al. identified a unique *O*-glycosite Thr 376 on the RBD that we could not identify in our study [65]. However, their experiment was conducted using the spike protein RBD expressed as a monomer, which may have prompted differentiation in the glycosylation pattern of the RBD [66]. The CTD1 and CTD2 also exhibited varying degrees of *O*-glycosylation. For the CTD1, a few of the variants were *O*-glycosylated in this domain including delta, iota, lambda, and omicron. Conversely, the CDT2 domain was *O*-glycosylated in all the variants except mu. The presence of a mutation on P681 has been found to decrease *O*-glycosylation on Thr678 in CTD2 [44]. Accordingly, our data indicates that *O*-glycosylation was not detected on Thr678 in VOCs except in the gamma variant. This discrepancy may be attributed to the absence of a mutation on residue P681 in gamma. 

Another intriguing observation in the glycosylation pattern observed in this analysis was the *O*-glycosylation proximal to *N*-glycosylation sites. Previously, Tian et al. reported *O*-glycosylation in proximity to glycosylated asparagine amino acid residue in wild-type SARS-CoV-2 spike protein, thereby suggesting the “O-Follow-N” rule [67]. We observed *O*-glycosylation on T124, T236, and S151 following potential *N*-glycosylation sites with the sequon NXS/T in some of the analyzed variants (Figure 2C). 

### 3.2. Microheterogeneity of Site-Specific O-Glycosylation

**Figure 3:**
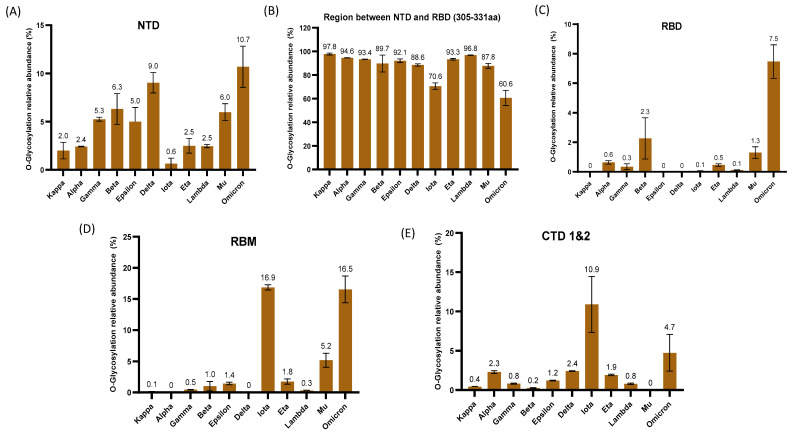
Distribution and quantification of *O*-glycosylation in domains and regions. The bar graphs represent the normalized total *O*-glycosylation abundance on (**A**) the NTD, (**B**) the region between NTD and RBD, (**C**) the RBD, (**D**) the RBM, and (**E**) the CTD 1 and 2 across the different variants. The normalized relative abundances are shown in percentage (%). The error bar represents the standard deviation (n = 3).

In the previous section, we described the qualitative differences in *O*-glycosylation between the domains and motifs of the S1 protein variants. It is well known that the site-glycosylation also presents large differences in glycan expression [68,69]. Therefore, we next quantitatively describe the heterogeneity of the identified glycosylation sites across the variants’ domains and regions. These include the domains NTD, RBD, RBM, CTD, and the region between the NTD and RBD designated as amino acid residues 305–331. Although the function of the NTD is not clear, it has been shown to be a potential target for therapeutics against SARS-CoV-2 [70]. SARS-CoV-2 binding by ACE2 occurs primarily at the RBD, a critical functional component within the S1 subunit [16,61,71]. The RBM in the RBD is the essential functional motif that facilitates interaction with the human angiotensin-converting enzyme (ACE2) [28]. Additionally, the CTD also functions as the RBD for the ACE2 entry receptor [26]. The data revealed that the relative abundances of *O*-glycosylation were not uniformly distributed across each domain or region, as shown in Figure 3A–E. Across all the variants, the region between the amino acids 305 to 331 contained the highest relative abundance of *O*-glycopeptides with a total average of 88%. It is noteworthy that the omicron variant showed the lowest *O*-glycosylation, with a relative abundance of approximately 61.0% in this region. The comparative analysis of *O*-glycosylation in the NTD revealed that all strains had levels below 11% of relative abundance, where omicron showed the largest abundance with 10.7% in this region. Remarkably, the relative abundance of the RBD domain was less than 3% in all strains except for omicron, with 7.5%. The relative abundance of *O*-glycosylation on the RBM functional motif was similar between iota and omicron with 16.9% and 16.5%, respectively. The *O*-glycosylation relative abundance in the rest of the analyzed strains was less than 6% in this motif. Considering the CTD’s involvement in the binding of the ACE2 receptor and the crucial function of the furin S1/S2 cleavage site in mediating the hydrolysis of S protein, we measured the *O*-glycosylation at the CTD 1 and 2 close to the cleavage site. In this domain, the iota strain had the highest relative abundance (10.9%), followed by omicron (4.7%). 

**Figure 4:**
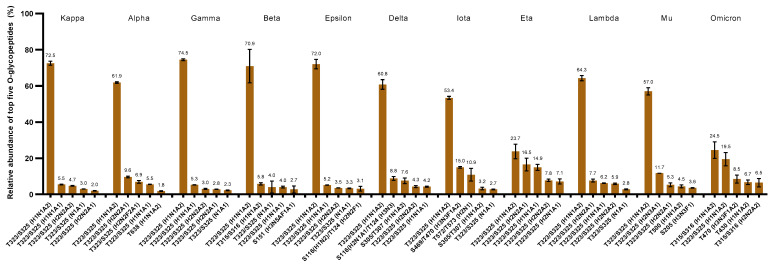
Quantification of the top five *O*-glycopeptides in each variant. The bar graph represents the normalized percentage relative abundance of the top five *O*-glycopeptides across the different variants. The error bar represents the standard deviation (n = 3).

Additionally, we analyzed the top five *O*-glycopeptides across all variants and determined that the most abundant *O*-glycan (H1N1A2) was observed on amino acid residue T323/S325 except in omicron (Figure 4). The omicron variant possesses this abundant glycan in position T315/S316. The disialylated core 1 *O*-glycan has been reported to be the most abundant in the wild type of SARS-CoV-2 S protein in many studies [46,72]. The single levels of this glycan varied between 75.0% and 53.4% relative abundance in almost all the evaluated variants (Figure 4). Conversely, the eta and omicron variants presented considerably lower relative abundances of this glycan H1N1A2 with 23.7% and 24.5%, respectively (Figure 4). In this case, both variants showed a uniform *O*-glycan distribution. This observation may be due to the increased number of *O*-glycosylation sites present in both variants. The relative abundances are spread across the multiple *O*-glycosylated sites. 

Because the RBD domain is the principal target of neutralizing antibodies, the bulk of SARS-CoV-2 specific antibodies is directed towards it [73]. Several mutations have occurred in the RBD of different SARS-CoV-2 variants that alter their binding by antibodies [74,75,76,77]. Reports from previous studies indicate that omicron with many mutations, especially in the spike protein, has the ability to evade the immune system, thereby increasing its infectivity and transmissibility [78,79]. Omicron’s RBD contains 15 substitutions, and two of these mutations (including G446S and G496S) have introduced new potential *O*-glycosylation sites. We identified *O*-glycosylation on mutation points G446S; however, this was considered ambiguous due to the lack of sufficient spectral evidence. Accordingly, our results indicated a significant increase in the *O*-glycosylation abundance of this domain in omicron (Figure 3C). These glycans may shield important epitopes aiding the immune evasion seen in omicron variants, thereby increasing its infectivity and transmissibility. Substitutions such as K417N and N501Y have been linked to immune evasion and increased infectivity [8,9,80]. In addition, the E484K mutation lowers antibody neutralization, which would favor escape mutations [81]. Point mutations involving substitution with electropositive amino acid residues such as Q493R and Q498R in the RBD of omicron have been shown to be critical for increased binding of its RBD to its ACE2 receptor [82,83]. The presence of these critical mutations in VOCs may explain their observed increase in infection. In addition to these mutations, the dense *O*-glycosylation observed in omicron may explain its ability to evolve into new variants of concern with enhanced transmissibility and antibody resistance [84]. Among the VOIs, iota has shown a dense distribution of *O*-glycosylation comparable to omicron on its RBM. However, it lacks mutations essential for immune escapism and increased infectivity. Contrary to the other strains of SARS-CoV-2 known to infect the lungs, omicron primarily targets the upper respiratory tract, resulting in milder symptoms and a greater transmission of the variant [85]. The expression of ACE2 in the upper respiratory tract is significantly low compared to the lungs [86,87]. The extensive *O*-glycosylation seen in the RBD of omicron may explain its ability to mainly infect the upper respiratory tract, which is characterized by poor expression of human ACE2.

*N*-glycosylation characterization and profiling in SARS-CoV-2 spike proteins have been conducted in many studies [29,30,31,88,89]. The significance of *N*-glycosylation in the pathogenicity of the virus has been demonstrated since it plays a pivotal role in facilitating viral attachment and entry into the host cell [35,36]. In addition, they can modulate both innate and adaptive immune responses in the virus, which ultimately impacts host recognition and infectivity [37,38]. A recent study investigated the *N*- and *O*-glycosylation profiling of SARS-CoV-2 variants of concern (VOCs) and reported changes in the *N*- and *O*-glycosylation profile [30]. However, the researchers were only able to confirm the presence of *O*-glycosite T323 which may be due to differences in the expression systems or expression methods.

Our results indicated that the omicron variant has a wide distribution of *O*-glycosylation on critical functional domains, which may explain its higher transmissibility and infectivity. Overall, the omicron variant possesses more *O*-glycosylation sites than other strains, with 23 unambiguous *O*-glycosylation sites. Moreover, combined with its extensive *O*-glycosylation, omicron’s *N*-glycosylation on the S1 subunit may ensure maximum glycan shielding of the protein potentially contributing to immune evasion.

### 3.3. Compositional Analysis of O-Glycoform Types

**Figure 5:**
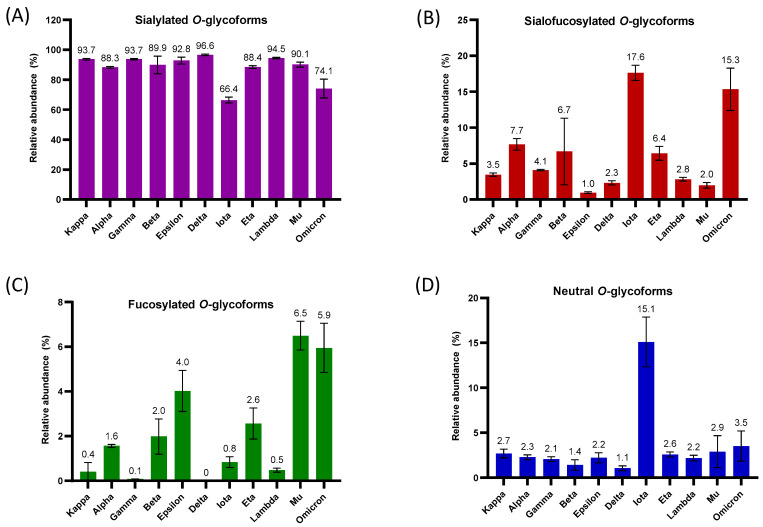
Distribution of *O*-glycoform types in different domains and regions across variants. (**A**) The bar graph represents the normalized percentage relative abundance of sialylated-only *O*-glycoforms across the different variants. (**B**) The bar graph represents the normalized percentage relative abundance of sialofucosylated *O*-glycoforms across the different variants. (**C**) The bar graph represents the normalized percentage relative abundance of fucosylated-only *O*-glycoforms across the different variants. (**D**) The bar graph represents the normalized percentage relative abundance of neutral *O*-glycoforms across the different variants. The error bar represents the standard deviation (n = 3).

Key components of glycosylation include glycoform types, which play a role in the interaction between the virus and the host [25]. Sialylated glycoforms have been demonstrated to be important in viral pathogenicity and transmission among species by playing crucial functions such as attachment and entry receptors for viruses in host cells [11,90]. Based on this information, it is imperative to carry out a comprehensive analysis of viral sialylation on SARS-CoV-2 variants. Herein, *O*-linked glycoform types generated from the S1 subunit of the SARS-CoV-2 strains were investigated and classified into sialylated, fucosylated, sialofucosylated, and other types, as depicted in Figure 5. 

To explore the different glycan types in the S1 proteins of SARS-CoV-2 variants, we computed the total normalized relative abundances of the different *O*-glycoform types (Figure 5A–D). According to our results, the sialylated *O*-glycans were the most abundant of all the variants, demonstrating the importance of sialylation in viral pathogenesis. The total average of sialylation across the investigated variants was 88%, while the iota and omicron strains showed lower relative abundances with 66.4% and 74.1%, respectively (Figure 5A). The top five *O*-glycans were the sialylated type, as can be observed in Figure 4. Of particular interest, the observed deficiency of sialylation of the iota and omicron variants was complemented with a significant increase of 17.6% and 15.3% in sialofucosylation, compared to other SARS-CoV-2 strains (Figure 5B). The most abundant sialofucosylated *O*-glycans were different across the variants. For instance, H2N2F2A1 was the most abundant in alpha, while H3N3F1A2 was the most abundant in omicron and iota (Figure 4). High expression of sialofucosylation has been linked to increased ACE2 binding and decreased sensitivity to neutralizing antibodies [91]. There is still a need for further research to determine the biological significance of *O*-sialofucosylation on the virology of SARS-CoV-2 variants. Additional results showed approximately 7% fucosylated *O*-glycoforms in mu and 6% in omicron, while the other variants exhibited ≤4% fucosylation (Figure 5C). The final *O*-glycoform type studied was the glycoforms without sialylation or fucosylation, termed neutral glycan types. In contrast to the other variants, iota had a much larger abundance of this type of *O*-glycosylation with 15.1% relative abundance, while others showed less than 4% (Figure 5D). The role that this glycoform type plays in iota deserves further investigation. 

Among the top five *O*-glycopeptides across all variants, the most prevalent *O*-glycoform in all variants was disialylated and located between NTD and RBD (Figure 4). Interestingly, among all the analyzed SARS-CoV-2 variants, the top five *O*-glycopeptides identified in only omicron were disialylated, with two of them present on the RBD (T430 and T470) functional domain (Figure 4). The most abundant glycopeptide in all variants was present in the region between the NTD and RBD. There is a need to explore sialylation on functional domains because the abundance of this glycopeptide was greater than 50% in all the examined SARS-CoV-2 S1 variants, except for eta and omicron (<25%).

To further explore the relevance of sialylation in SARS-CoV-2 variant pathogenesis and interspecies transmission, we investigated the total sialylation on the RBD and RBM functional domains in the S1 proteins of the variants. Notably, omicron showed 7.5% sialylation abundance in the RBD; in comparison, all other variants showed less than 1% sialylation in the RBD (Supplementary Figure S2). Prior studies have indicated that the increased sialylation observed in the omicron variant may potentially augment its ability to evade neutralization [91]. Roberts et al. have also reported increased sialylation in the RBD of WT and SARS-CoV-2 variants [65]. In the RBM, there was a comparable sialylation abundance in omicron and iota with 15.5% and 17.8%, respectively, while the others showed less than 6% sialylation in this functional motif (Supplementary Figure S3). This is due to the higher sialylation abundance in the region between NTD and RBD in the other variants that were analyzed.

It is still unknown how the essential amino acid point mutation interacts with proximal *O*-linked glycans in various SARS-CoV-2 variants. According to our results, only the omicron variant exhibited six *O*-glycoforms on position T500 next to the immune escape mutation point N501Y in the RBM, while the other strains had just one *O*-glycoform or none (Figure 2C). More interestingly, the six *O*-glycoforms found on the T500 position in the omicron variant were sialylated, further implicating sialylation in the infectivity and transmissibility of this variant. It is important to reiterate that the RBM on the RBD is the functional motif that interacts directly with the human ACE2 for viral entry [28]. Thus, this type of glycosylation may influence the viral life cycle and host interaction. 

After evaluating the abundance of the different *O*-glycoform types, we subsequently employed heat maps to illustrate the normalized abundances of individual glycoforms on the identified glycosites in all analyzed variants of SARS-CoV-2, as depicted in Supplementary Figures S4–S14. 

### 3.4. Differentially Expressed O-Glycosylation among SARS-CoV-2 S1 Protein Variants

**Figure 6:**
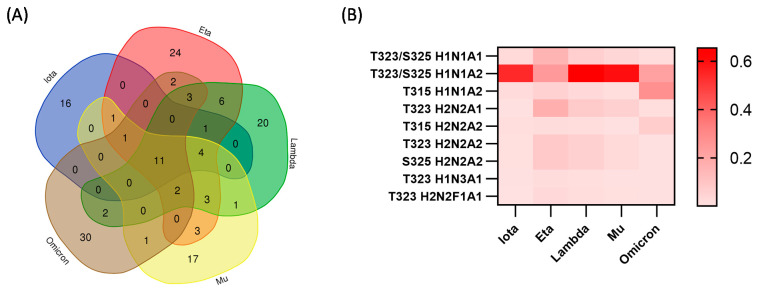
Comparative analysis of microheterogeneity among five variants. (**A**) Venn plot showing the shared and unique *O*-glycopeptides among iota, eta, lambda, mu, and omicron. (**B**) Heatmap of *O*-glycopeptides common to iota, eta, lambda, mu, and omicron illustrating relative abundance.

In this section, we focus our evaluation on the variants with a denser distribution of *O*-glycosylation on the S1 protein: iota, eta, lambda, mu, and omicron. Interestingly, these variants showed significant differences in the microheterogeneity of glycoforms present on the identified glycosites. To provide useful analytical information, we investigated how these five variants differ in terms of *O*-glycoforms. The results showed 16, 24, 20, 17, and 30 unique *O*-glycopeptides for iota, eta, lambda, mu, and omicron SARS-CoV-2 S1 proteins, respectively. Eleven *O*-glycopeptides were found in common among the evaluated variants (Figure 6A). The list of unique and shared *O*-glycopeptides present in these variants is shown in Supplementary Table S3. It is worth noting that the common *O*-glycopeptides were present in the region between the amino acids 305 to 331. The region observed between the NTD and RBD was the most glycosylated region in all the SARS-CoV-2 S1 proteins analyzed in this approach. Although the eta variant showed the largest number of *O*-linked glycans, omicron had the largest number of unique *O*-glycopeptides prompted by its higher number of identified *O*-glycosites. This information suggests a greater complexity in the *O*-glycosylation of omicron compared to the other variants.

The relative abundances of the common *O*-glycopeptides shared by the SARS-CoV-2 variants were plotted as a heatmap (Figure 6B). Where the SARS-CoV-2 variants are shown on the x-axis, the glycosylation sites and *O*-linked glycans are shown along the y-axis for comparison of *O*-glycoform expressions. The results showed a significantly higher abundance of H1N1A2 on T323/S325 of the variants iota, lambda, and mu. Conversely, the eta and omicron variants showed a more uniform *O*-glycan expression, a difference that was also demonstrated in Figure 4. The expression of T315 H1N1A2 may be comparable in the iota, eta, lambda, and mu variants, but it is significantly more abundant in omicron. This comparison demonstrates that common *O*-glycoforms are shared by the SARS-CoV-2 variants, but also highlights the variation in their expressions across the variants. 

### 3.5. HCD-EThcD Dissociation of Tryptic-IMPa Digested O-Glycopeptides

**Figure 7:**
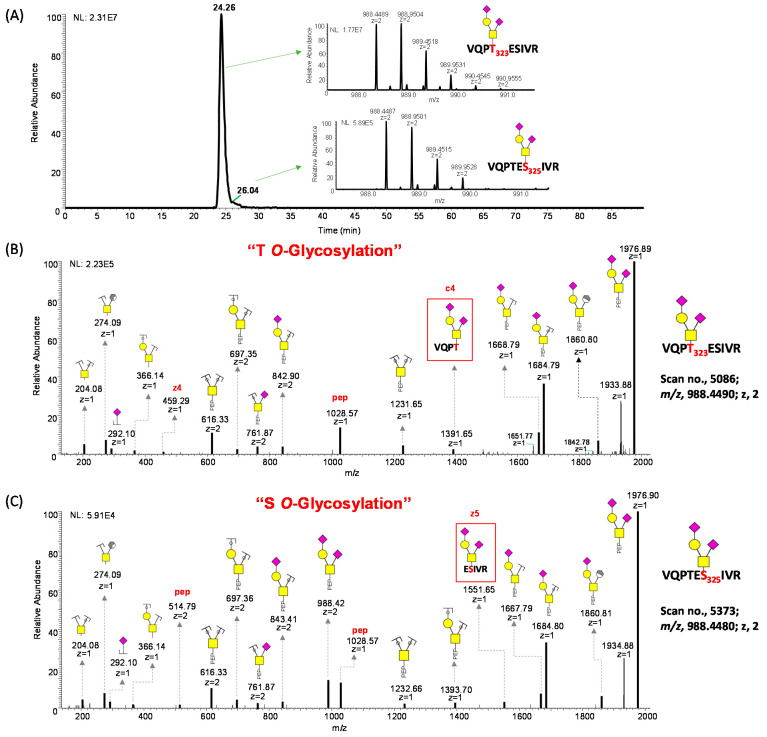
EThcD tandem mass spectra of a tryptic/IMPa digested glycopeptide confirming the occupancy of both T323 and S325 *O*-glycosylation sites in beta variant (H15). (A) An Extracted Ion Chromatogram (EIC) of isobaric *O*-glycopeptide VQPTESIVR-H1N1A1. The insets represent the MS1 spectra for the isobars. The c4 fragment in (**B**) shows that T323 is glycosylated and the z5 fragment in (**C**) shows that S325 is glycosylated.

Unlike *N*-glycosylation, the motifs observed for *O*-glycosylation are more complicated. *O*-glycoproteomic analysis is challenging due to differences in glycoforms attached to a glycosylation site and the site occupancy [27,54]. Therefore, the digestion method and instrument methodology employed have a significant impact on the number of identified *O*-glycosylation sites and the quality of MS/MS spectra that will be generated [69,92]. Another difficulty associated with the study of *O*-glycosites using glycoproteomic analysis is the fact that several of these sites are relatively close to one another. Thus, often the final glycopeptides contain more than one glycosylation site occupied with the same or different glycans. A clear example is the heavily glycosylated T323/S325 in the S-protein of SARS-CoV-2, which has posed a significant challenge in deciphering the specific localization of glycosylation in studies conducted by many groups [27,29]. Studies have shown that HCD does not provide a confident assignment of site localization for multiply glycosylated peptides [59,69,93]. To overcome this problem, coupled with double digestion using tryptic and IMPa digestion, we employed an HCD–EThcD pair dissociation method for selective backbone fragmentation to aid the confident assignment of *O*-linked glycans on adjacent S and T present on the same backbone. In this technique, HCD is first used to generate a spectrum that facilitates accurate identification of peptide backbone fragments without localization of the glycan. However, the precursor mass in the HCD spectrum corresponds to a combination of the peptide and the *O*-glycans. Subsequently, the paired EThcD spectrum allows for the site-specific localization of *O*-glycosylation even in multiply glycosylated peptides [59,93,94]. The reporter ions produced by HCD are systematically merged with the signature fragments necessary for *O*-glycosite localization generated by EThCD in a sequential manner [95]. The utilization of this integrated fragmentation technique has the capability to generate a broader range of fragment ions, hence enhancing the analysis of site-specific glycosylation. Moreover, the resulting spectra exhibit a high level of quality [96]. With this novel approach, we have been able to show that both T323 and S325 are *O*-glycosylated. Figure 7A shows the EICs of the positional isomers of the *O*-glycopeptides (VQPT_323_ESIVR) + H1N1A2 and (VQPTES_325_IVR) + H1N1A2. Figure 7B shows the mass spectra of the *O*-glycopeptide (VQPT_323_ESIVR) + H1N1A2. The C4 fragment “VQPT323 + H1N1A2” with an *m*/*z* value of 1391.65 shows that T323 is occupied with the *O*-glycan H1N1A2, and a z4 fragment with the rest of the peptide backbone. Figure 7C shows the mass spectra of the *O*-glycopeptide (VQPTES325IVR) + H1N1A2. In this case, the fragment z5 “ES325IVR + H1N1A2” with an *m*/*z* value of 1550.65 shows that S325 is occupied with the *O*-glycan H1N1A2. Although our instrumentation generated MS/MS spectra for qualitative identification of these isobaric *O*-glycopeptide ions, we were unable to obtain a good resolution of the peaks with the employed analytical technique. We have also shown the same peptide “VQPTESIVR” that is doubly glycosylated with different glycans confidently assigned to the glycosylation sites T323 and S325, as shown in Supplementary Figure S15. The z5 fragment confirms the linkage of H1N2A1 to S325, while the c6 fragment shows the linkage of H1N1 to T323 and H1N2A1 to S325.

### 3.6. O-Glycosylation at Points of Mutation

**Figure 8:**
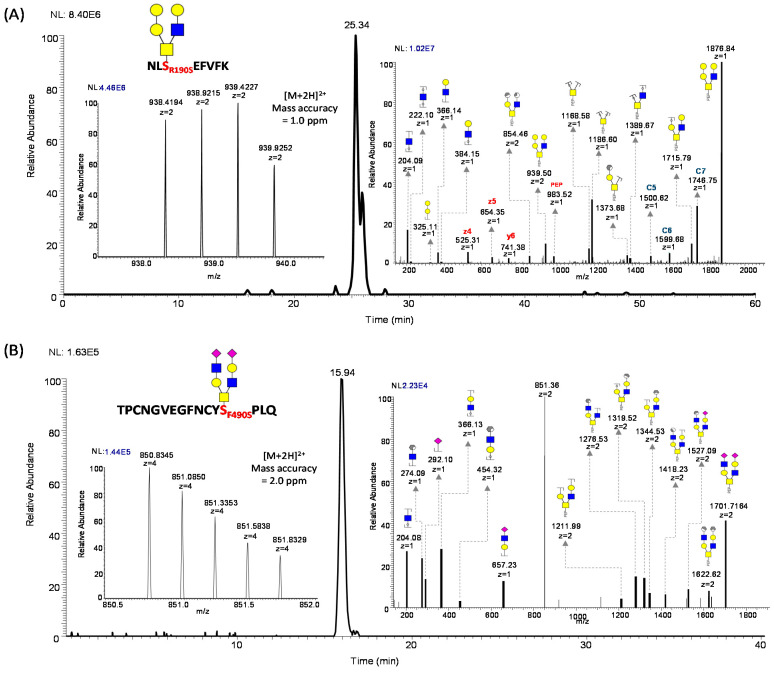
Localization of *O*-glycopeptides on mutation hotspots. The extracted ion chromatogram of *O*-glycopeptide on (**A**) R190S mutation present in gamma variant, (**B**) F490S mutation present in lambda variant.

The mutations in the sequences of the different SARS-CoV-2 variants may alter the conformation of the proteins, which can impact the accessibility of *O*-glycosylation enzymes [97]. This could increase the probability of variant-dependent heterogeneous glycosylation, altering the glycosylation pattern in different variants. To the best of our knowledge, we will be the first to report *O*-glycosylation at points of mutations in SAR-CoV-2 variants. According to our results, the R190S mutation observed in the gamma variant, and F490S in the lambda variant, are shown to be *O*-glycosylated with spectral evidence (Figure 8). In addition, we identified *O*-glycosylation at G446S mutation in omicron, but without confident spectral evidence (Supplementary Table S4). The implications of these changes in the viral life cycle and interaction with the host call for further exploration.

## 4. Conclusions

SARS-CoV-2 remains a major health concern despite numerous scientific efforts to contain it. The changes in glycosylation patterns due to mutations in the S protein of SARS-CoV-2 variants necessitate thorough investigation. Here, we have demonstrated site-specific macro- and microheterogeneity in the pattern of *O*-glycosylation across 11 SARS-CoV-2 variants. We examined *O*-glycosylation in various functional domains to assess the effect of mutation on the glycosylation pattern in the S1 protein of SARS-CoV-2 variants. Our findings are important to understand how these glycosylation changes influence viral pathogenicity, tropism, immunological evasion, and virus-host interaction, all of which are critical in the development of a robust activity vaccine for managing the emerging SARS-CoV-2 variants of concern. Furthermore, we discovered *O*-glycosylation at mutation hotspots for the first time. Finally, whether *N*-glycosylation can complement the observed *O*-glycosylation for maximum glycan shielding should be further investigated. 

## Figures and Tables

**Table 1:** List of the SARS-CoV-2 variants showing the S1 accession ID, name, lineage, and list of mutations.

SARS-CoV-2 S1 Accession ID *	Variant Name	Lineage	Mutations/Deletions/Insertions
40591-V08H12	Alpha	B.1.1.7	HV69-70 deletion, Y144 deletion, N501Y, A570D, D614G, P681H
40591-V08H14	Gamma	P.1	L18F, T20N, P26S, D138Y, R190S, K417T, E484K, N501Y, D614G, H655Y
40591-V08H15	Beta	B.1.351	L18F, D80A, D215G, LAL242-244 deletion, R246I, K417N, E484K, N501Y, D614G
40591-V08H17	Epsilon	B.1.427	W152C, L452R, D614G
40591-V08H23	Delta	B.1.617.2	T19R, G142D, E156G, 157–158 deletion, L452R, T478K, D614G, P681R
40591-V08H28	Iota	B.1.526	L5F, T95I, D253G, S477N, E484K, D614G
40591-V08H29	Eta	B.1.525	Q52R, A67V, 69–70 deletion, 144 deletion, E484K, D614G, Q677H
40591-V08H32	Lambda	C.37	G75V, T76I, RSYLTPG246-252 deletion, D253N, L452Q, F490S, D614G
40591-V08H38	Mu	B.1.621	T95I, Y144S, Y145N, R346K, E484K, N501Y, D614G, P681H
40591-V08H41	Omicron	B.1.1.529	A67V, Δ69-70, T95I, G142D/Δ143-145, Δ211I/L212I, ins214EPE, G339D, S371L, S373P, S375F, K417N, N440K, G446S, S477N, T478K, E484A, Q493R, G496S, Q498R, N501Y, Y505H, T547K, D614G, H655Y, N679K, P681H
40591-V08H1-B	Kappa	B.1.617.1	T95I, G142D, E154K, L452R, E484Q, D614G, P681R

* Vendor part numbers (Sino Biologicals Inc.).

## Data Availability

The mass spectrometry proteomics data have been deposited to the ProteomeXchange Consortium via the PRIDE partner repository with the dataset identifier PXD041282 and 10.6019/PXD041282.

## Supplementary Materials

The following supporting information can be downloaded at: https://www.mdpi.com/article/10.3390/biom13101467/s1, Figure S1: Amino acid sequences and purity of the acquired S1 glycoproteins of eleven strains of SARS-CoV-2 that were expressed in human embryonic kidney 293 (HEK293) cells; Figure S2: Sialylation relative abundance on the receptor binding domain (RBD) of the variants S1 glycoproteins; Figure S3: Sialylation relative abundance on the receptor binding motif (RBM) of the variants S1 glycoproteins; Figure S4: Heatmap showing the relative abundance of individual *O*-glycoforms identified in alpha; Figure S5: Heatmap showing the relative abundance of individual *O*-glycoforms identified in beta; Figure S6: Heatmap showing the relative abundance of individual *O*-glycoforms identified in gamma; Figure S7: Heatmap showing the relative abundance of individual *O*-glycoforms identified in delta; Figure S8: Heatmap showing the relative abundance of individual *O*-glycoforms identified in epsilon; Figure S9: Heatmap showing the relative abundance of individual *O*-glycoforms identified in kappa; Figure S10: Heatmap showing the relative abundance of individual *O*-glycoforms identified in iota; Figure S11: Heatmap showing the relative abundance of individual *O*-glycoforms identified in eta; Figure S12: Heatmap showing the relative abundance of individual *O*-glycoforms identified in lambda; Figure S13: Heatmap showing the relative abundance of individual *O*-glycoforms identified in mu; Figure S14: Heatmap showing the relative abundance of individual *O*-glycoforms identified in omicron; Figure S15: EThcD tandem mass spectra of a tryptic/IMPa digested glycopeptide confirming the occupancy of both T323 and S325 *O*-glycosylation sites; Data extracted from eta variant (H29) with Scan no., 3354; *m*/*z*, 751.6761; z, 3; Figure S16: EThcD tandem mass spectra of a tryptic/IMPa digested glycopeptide confirming the occupancy of a mutation point (R190S) in Gamma; Table S1: *O*-glycopeptides identified by Byonic; Table S2: *O*-glycopeptides identified by MetaMorpheus; Table S3: Venn plot results of unique and shared *O*-glycopeptides among five variants. Table S4: Ambiguous *O*-glycopeptides identified on G446S in omicron.
