# Supplementary material for: Variations in O-Glycosylation Patterns Influence Viral Pathogenicity, Infectivity, and Transmissibility in SARS-CoV-2 Variants
Source: Biomolecules. 2023 Sep 29;13(10):1467. doi: 10.3390/biom13101467 (PMC10604390; doi:10.3390/biom13101467)

Supplementary Information

Variations in *O*-glycosylation Patterns Influence Viral Pathogenicity, Infectivity, and Transmissibility in SARS-CoV-2 Variants

Sherifdeen Onigbinde [1]‡, Cristian D. Gutierrez Reyes [1]‡, Mojibola Fowowe [1], Oluwatosin Daramola [1], Mojgan Atashi [1], Andrew I. Bennett [1], and Yehia Mechref [1,*]

[1]Department of Chemistry and Biochemistry, Texas Tech University, Lubbock, TX 79409-1061

‡ These authors contribute equally

***Corresponding Author**

Department of Chemistry and Biochemistry

Texas Tech University

Lubbock, TX 79409-1061

Email: Yehia.Mechref@ttu.edu

Tel: 806-742-3059

Fax: 806-742-1289

Table of Contents:

Supplementary Figures:

Supplementary Figure S1. Amino acid sequences and purity of the acquired S1 glycoproteins of eleven strains of SARS-CoV-2 that were expressed in human embryonic kidney 293 (HEK293) cells.

Supplementary Figure S2. Sialylation relative abundance on the receptor binding domain (RBD) of the variants S1 glycoproteins.

Supplementary Figure S3. Sialylation relative abundance on the receptor binding motif (RBM) of the variants S1 glycoproteins.

Supplementary Figure S4. Heatmap showing the relative abundance of individual *O*-glycoforms identified in **alpha.**

Supplementary Figure S5. Heatmap showing the relative abundance of individual *O*-glycoforms identified in **beta.**

Supplementary Figure S6. Heatmap showing the relative abundance of individual *O*-glycoforms identified in **gamma**.

Supplementary Figure S7. Heatmap showing the relative abundance of individual *O*-glycoforms identified in **delta.**

Supplementary Figure S8. Heatmap showing the relative abundance of individual *O*-glycoforms identified in **epsilon.**

Supplementary Figure S9. Heatmap showing the relative abundance of individual *O*-glycoforms identified in **kappa.**

Supplementary Figure S10. Heatmap showing the relative abundance of individual *O*-glycoforms identified in **iota.**

Supplementary Figure S11. Heatmap showing the relative abundance of individual *O*-glycoforms identified in **eta.**

Supplementary Figure S12. Heatmap showing the relative abundance of individual *O*-glycoforms identified in **lambda.**

Supplementary Figure S13. Heatmap showing the relative abundance of individual *O*-glycoforms identified in **mu.**

Supplementary Figure S14. Heatmap showing the relative abundance of individual *O*-glycoforms identified in **omicron.**

Supplementary Figure S15. EThcD tandem mass spectra of a tryptic/IMPa digested glycopeptide confirming the occupancy of both T323 and S325 *O*-glycosylation sites. Data extracted from eta variant (H29) with **Scan no., 3354; m/z, 751.6761; z, 3.**

Supplementary Figure S16. EThcD tandem mass spectra of a tryptic/IMPa digested glycopeptide confirming the occupancy of a mutation point (R190S) in Gamma.

Supplementary Tables:

Supplementary Table S1: List of *O*-glycopeptides identified in the different variants by Byonic software.

Supplementary Table S2: List of *O*-glycopeptides identified in the different variants by MetaMorpheus software.

Supplementary Table S3: Venn plot results showing the list of *O*-glycopeptides common and unique to Iota, Eta, Lambda, Mu, and Omicron.

Supplementary Table S4: List of O-glycopeptides on G446S mutation in Omicron.

Supplementary Figure S1

1 100
Alpha MFVFLVLLPL VSSQCVNLTT RTQLPPAYTN SFTRGVYYPD KVFRSSVLHS TQDLFLPFFS NVTWFHAI-- SGTNGTKRFD NPVLPFNDGV YFASTEKSNI
Beta MFVFLVLLPL VSSQCVNFTT RTQLPPAYTN SFTRGVYYPD KVFRSSVLHS TQDLFLPFFS NVTWFHAIHV SGTNGTKRFA NPVLPFNDGV YFASTEKSNI
Gamma MFVFLVLLPL VSSQCVNFTN RTQLPSAYTN SFTRGVYYPD KVFRSSVLHS TQDLFLPFFS NVTWFHAIHV SGTNGTKRFD NPVLPFNDGV YFASTEKSNI
Delta MFVFLVLLPL VSSQCVNLRT RTQLPPAYTN SFTRGVYYPD KVFRSSVLHS TQDLFLPFFS NVTWFHAIHV SGTNGTKRFD NPVLPFNDGV YFASTEKSNI
Epsilon MFVFLVLLPL VSSQCVNLTT RTQLPPAYTN SFTRGVYYPD KVFRSSVLHS TQDLFLPFFS NVTWFHAIHV SGTNGTKRFD NPVLPFNDGV YFASTEKSNI
Kappa MFVFLVLLPL VSSQCVNLTT RTQLPPAYTN SFTRGVYYPD KVFRSSVLHS TQDLFLPFFS NVTWFHAIHV SGTNGTKRFD NPVLPFNDGV YFASIEKSNI
Iota MFVFFVLLPL VSSQCVNLTT RTQLPPAYTN SFTRGVYYPD KVFRSSVLHS TQDLFLPFFS NVTWFHAIHV SGTNGTKRFD NPVLPFNDGV YFASIEKSNI
Eta MFVFLVLLPL VSSQCVNLTT RTQLPPAYTN SFTRGVYYPD KVFRSSVLHS TRDLFLPFFS NVTWFHVI-- SGTNGTKRFD NPVLPFNDGV YFASTEKSNI
Lambda MFVFLVLLPL VSSQCVNLTT RTQLPPAYTN SFTRGVYYPD KVFRSSVLHS TQDLFLPFFS NVTWFHAIHV SGTNVIKRFD NPVLPFNDGV YFASTEKSNI
Mu MFVFLVLLPL VSSQCVNLTT RTQLPPAYTN SFTRGVYYPD KVFRSSVLHS TQDLFLPFFS NVTWFHAIHV SGTNGTKRFD NPVLPFNDGV YFASIEKSNI
Omicron MFVFLVLLPL VSSQCVNLTT RTQLPPAYTN SFTRGVYYPD KVFRSSVLHS TQDLFLPFFS NVTWFHVI-- SGTNGTKRFD NPVLPFNDGV YFASIEKSNI

101 200
Alpha IRGWIFGTTL GSKTQSLLIV NNATNVVIKV CEFQFCNDPF LGV-YHKNNK SWMESEFRVY SSANNCTFEY VSQPFLMDLE GKQGNFKNLR EFVFKNIDGY
Beta IRGWIFGTTL GSKTQSLLIV NNATNVVIKV CEFQFCNDPF LGVYYHKNNK SWMESEFRVY SSANNCTFEY VSQPFLMDLE GKQGNFKNLR EFVFKNIDGY
Gamma IRGWIFGTTL GSKTQSLLIV NNATNVVIKV CEFQFCNYPF LGVYYHKNNK SWMESEFRVY SSANNCTFEY VSQPFLMDLE GKQGNFKNLS EFVFKNIDGY
Delta IRGWIFGTTL GSKTQSLLIV NNATNVVIKV CEFQFCNDPF LDVYYHKNNK SWMESG--VY SSANNCTFEY VSQPFLMDLE GKQGNFKNLR EFVFKNIDGY
Epsilon IRGWIFGTTL GSKTQSLLIV NNATNVVIKV CEFQFCNDPF LGVYYHKNNK SCMESEFRVY SSANNCTFEY VSQPFLMDLE GKQGNFKNLR EFVFKNIDGY
Kappa IRGWIFGTTL DSKTQSLLIV NNATNVVIKV CEFQFCNDPF LDVYYHKNNK SWMKSEFRVY SSANNCTFEY VSQPFLMDLE GKQGNFKNLR EFVFKNIDGY
Iota IRGWIFGTTL GSKTQSLLIV NNATNVVIKV CEFQFCNDPF LGVYYHKNNK SWMESEFRVY SSANNCTFEY VSQPFLMDLE GKQGNFKNLR EFVFKNIDGY
Eta IRGWIFGTTL GSKTQSLLIV NNATNVVIKV CEFQFCNDPF LGV-YHKNNK SWMESEFRVY SSANNCTFEY VSQPFLMDLE GKQGNFKNLR EFVFKNIDGY
Lambda IRGWIFGTTL GSKTQSLLIV NNATNVVIKV CEFQFCNDPF LGVYYHKNNK SWMESEFRVY SSANNCTFEY VSQPFLMDLE GKQGNFKNLR EFVFKNIDGY
Mu IRGWIFGTTL GSKTQSLLIV NNATNVVIKV CEFQFCNDPF LGVSNHKNNK SWMESEFRVY SSANNCTFEY VSQPFLMDLE GKQGNFKNLR EFVFKNIDGY
Omicron IRGWIFGTTL GSKTQSLLIV NNATNVVIKV CEFQFCNDPF LDVYYHKNNK SWMESEFRVY SSANNCTFEY VSQPFLMDLE GKQGNFKNLR EFVFKNIDGY

201 300
Alpha FKIYSKHTPI NLVRDLPQGF SALEPLVDLP IGINITRFQT LLALHRSYLT PGDSSSGWTA GAAAYYVGYL QPRTFLLKYN ENGTITDAVD CALDPLSETK
Beta FKIYSKHTPI NLVRGLPQGF SALEPLVDLP IGINITRFQT L---HISYLT PGDSSSGWTA GAAAYYVGYL QPRTFLLKYN ENGTITDAVD CALDPLSETK
Gamma FKIYSKHTPI NLVRDLPQGF SALEPLVDLP IGINITRFQT LLALHRSYLT PGDSSSGWTA GAAAYYVGYL QPRTFLLKYN ENGTITDAVD CALDPLSETK
Delta FKIYSKHTPI NLVRDLPQGF SALEPLVDLP IGINITRFQT LLALHRSYLT PGDSSSGWTA GAAAYYVGYL QPRTFLLKYN ENGTITDAVD CALDPLSETK
Epsilon FKIYSKHTPI NLVRDLPQGF SALEPLVDLP IGINITRFQT LLALHRSYLT PGDSSSGWTA GAAAYYVGYL QPRTFLLKYN ENGTITDAVD CALDPLSETK
Kappa FKIYSKHTPI NLVRDLPQGF SALEPLVDLP IGINITRFQT LLALHRSYLT PGDSSSGWTA GAAAYYVGYL QPRTFLLKYN ENGTITDAVD CALDPLSETK
Iota FKIYSKHTPI NLVRDLPQGF SALEPLVDLP IGINITRFQT LLALHRSYLT PGGSSSGWTA GAAAYYVGYL QPRTFLLKYN ENGTITDAVD CALDPLSETK
Eta FKIYSKHTPI NLVRDLPQGF SALEPLVDLP IGINITRFQT LLALHRSYLT PGDSSSGWTA GAAAYYVGYL QPRTFLLKYN ENGTITDAVD CALDPLSETK
Lambda FKIYSKHTPI NLVRDLPQGF SALEPLVDLP IGINITRFQT LLALHR---- --NSSSGWTA GAAAYYVGYL QPRTFLLKYN ENGTITDAVD CALDPLSETK
Mu FKIYSKHTPI NLVRDLPQGF SALEPLVDLP IGINITRFQT LLALHRSYLT PGDSSSGWTA GAAAYYVGYL QPRTFLLKYN ENGTITDAVD CALDPLSETK
EPE
Omicron FKIYSKHTPI NIVRDLPQGF SALEPLVDLP IGINITRFQT LLALHRSYLT PGDSSSGWTA GAAAYYVGYL QPRTFLLKYN ENGTITDAVD CALDPLSETK

301 400
Alpha CTLKSFTVEK GIYQTSNFRV QPTESIVRFP NITNLCPFGE VFNATRFASV YAWNRKRISN CVADYSVLYN SASFSTFKCY GVSPTKLNDL CFTNVYADSF
Beta CTLKSFTVEK GIYQTSNFRV QPTESIVRFP NITNLCPFGE VFNATRFASV YAWNRKRISN CVADYSVLYN SASFSTFKCY GVSPTKLNDL CFTNVYADSF
Gamma CTLKSFTVEK GIYQTSNFRV QPTESIVRFP NITNLCPFGE VFNATRFASV YAWNRKRISN CVADYSVLYN SASFSTFKCY GVSPTKLNDL CFTNVYADSF
Delta CTLKSFTVEK GIYQTSNFRV QPTESIVRFP NITNLCPFGE VFNATRFASV YAWNRKRISN CVADYSVLYN SASFSTFKCY GVSPTKLNDL CFTNVYADSF
Epsilon CTLKSFTVEK GIYQTSNFRV QPTESIVRFP NITNLCPFGE VFNATRFASV YAWNRKRISN CVADYSVLYN SASFSTFKCY GVSPTKLNDL CFTNVYADSF
Kappa CTLKSFTVEK GIYQTSNFRV QPTESIVRFP NITNLCPFGE VFNATRFASV YAWNRKRISN CVADYSVLYN SASFSTFKCY GVSPTKLNDL CFTNVYADSF
Iota CTLKSFTVEK GIYQTSNFRV QPTESIVRFP NITNLCPFGE VFNATRFASV YAWNRKRISN CVADYSVLYN SASFSTFKCY GVSPTKLNDL CFTNVYADSF
Eta CTLKSFTVEK GIYQTSNFRV QPTESIVRFP NITNLCPFGE VFNATRFASV YAWNRKRISN CVADYSVLYN SASFSTFKCY GVSPTKLNDL CFTNVYADSF
Lambda CTLKSFTVEK GIYQTSNFRV QPTESIVRFP NITNLCPFGE VFNATRFASV YAWNRKRISN CVADYSVLYN SASFSTFKCY GVSPTKLNDL CFTNVYADSF
Mu CTLKSFTVEK GIYQTSNFRV QPTESIVRFP NITNLCPFGE VFNATKFASV YAWNRKRISN CVADYSVLYN SASFSTFKCY GVSPTKLNDL CFTNVYADSF
Omicron CTLKSFTVEK GIYQTSNFRV QPTESIVRFP NITNLCPFDE VFNATRFASV YAWNRKRISN CVADYSVLYN LAPFFTFKCY GVSPTKLNDL CFTNVYADSF

401 500
Alpha VIRGDEVRQI APGQTGKIAD YNYKLPDDFT GCVIAWNSNN LDSKVGGNYN YLYRLFRKSN LKPFERDIST EIYQAGSTPC NGVEGFNCYF PLQSYGFQPT
Beta VIRGDEVRQI APGQTGNIAD YNYKLPDDFT GCVIAWNSNN LDSKVGGNYN YLYRLFRKSN LKPFERDIST EIYQAGSTPC NGVKGFNCYF PLQSYGFQPT
Gamma VIRGDEVRQI APGQTGTIAD YNYKLPDDFT GCVIAWNSNN LDSKVGGNYN YLYRLFRKSN LKPFERDIST EIYQAGSTPC NGVKGFNCYF PLQSYGFQPT
Delta VIRGDEVRQI APGQTGKIAD YNYKLPDDFT GCVIAWNSNN LDSKVGGNYN YRYRLFRKSN LKPFERDIST EIYQAGSKPC NGVEGFNCYF PLQSYGFQPT
Epsilon VIRGDEVRQI APGQTGKIAD YNYKLPDDFT GCVIAWNSNN LDSKVGGNYN YRYRLFRKSN LKPFERDIST EIYQAGSTPC NGVEGFNCYF PLQSYGFQPT
Kappa VIRGDEVRQI APGQTGKIAD YNYKLPDDFT GCVIAWNSNN LDSKVGGNYN YRYRLFRKSN LKPFERDIST EIYQAGSTPC NGVQGFNCYF PLQSYGFQPT
Iota VIRGDEVRQI APGQTGKIAD YNYKLPDDFT GCVIAWNSNN LDSKVGGNYN YLYRLFRKSN LKPFERDIST EIYQAGNTPC NGVKGFNCYF PLQSYGFQPT
Eta VIRGDEVRQI APGQTGKIAD YNYKLPDDFT GCVIAWNSNN LDSKVGGNYN YLYRLFRKSN LKPFERDIST EIYQAGSTPC NGVKGFNCYF PLQSYGFQPT
Lambda VIRGDEVRQI APGQTGKIAD YNYKLPDDFT GCVIAWNSNN LDSKVGGNYN YQYRLFRKSN LKPFERDIST EIYQAGSTPC NGVEGFNCYS PLQSYGFQPT
Mu VIRGDEVRQI APGQTGKIAD YNYKLPDDFT GCVIAWNSNN LDSKVGGNYN YLYRLFRKSN LKPFERDIST EIYQAGSTPC NGVKGFNCYF PLQSYGFQPT
Omicron VIRGDEVRQI APGQTGNIAD YNYKLPDDFT GCVIAWNSNK LDSKVSGNYN YLYRLFRKSN LKPFERDIST EIYQAGNKPC NGVAGFNCYF PLRSYSFRPT

501 600
Alpha YGVGYQPYRV VVLSFELLHA PATVCGPKKS TNLVKNKCVN FNFNGLTGTG VLTESNKKFL PFQQFGRDID DTTDAVRDPQ TLEILDITPC SFGGVSVITP
Beta YGVGYQPYRV VVLSFELLHA PATVCGPKKS TNLVKNKCVN FNFNGLTGTG VLTESNKKFL PFQQFGRDIA DTTDAVRDPQ TLEILDITPC SFGGVSVITP Gamma
YGVGYQPYRV VVLSFELLHA PATVCGPKKS TNLVKNKCVN FNFNGLTGTG VLTESNKKFL PFQQFGRDIA DTTDAVRDPQ TLEILDITPC SFGGVSVITP Delta
NGVGYQPYRV VVLSFELLHA PATVCGPKKS TNLVKNKCVN FNFNGLTGTG VLTESNKKFL PFQQFGRDIA DTTDAVRDPQ TLEILDITPC SFGGVSVITP Epsilon
NGVGYQPYRV VVLSFELLHA PATVCGPKKS TNLVKNKCVN FNFNGLTGTG VLTESNKKFL PFQQFGRDIA DTTDAVRDPQ TLEILDITPC SFGGVSVITP Kappa
NGVGYQPYRV VVLSFELLHA PATVCGPKKS TNLVKNKCVN FNFNGLTGTG VLTESNKKFL PFQQFGRDIA DTTDAVRDPQ TLEILDITPC SFGGVSVITP Iota
NGVGYQPYRV VVLSFELLHA PATVCGPKKS TNLVKNKCVN FNFNGLTGTG VLTESNKKFL PFQQFGRDIA DTTDAVRDPQ TLEILDITPC SFGGVSVITP Eta
NGVGYQPYRV VVLSFELLHA PATVCGPKKS TNLVKNKCVN FNFNGLTGTG VLTESNKKFL PFQQFGRDIA DTTDAVRDPQ TLEILDITPC SFGGVSVITP Lambda
NGVGYQPYRV VVLSFELLHA PATVCGPKKS TNLVKNKCVN FNFNGLTGTG VLTESNKKFL PFQQFGRDIA DTTDAVRDPQ TLEILDITPC SFGGVSVITP
Mu YGVGYQPYRV VVLSFELLHA PATVCGPKKS TNLVKNKCVN FNFNGLTGTG VLTESNKKFL PFQQFGRDIA DTTDAVRDPQ TLEILDITPC SFGGVSVITP Omicron
YGVGHQPYRV VVLSFELLHA PATVCGPKKS TNLVKNKCVN FNFNGLKGTG VLTESNKKFL PFQQFGRDIA DTTDAVRDPQ TLEILDITPC SFGGVSVITP

601 685
Alpha GTNTSNQVAV LYQGVNCTEV PVAIHADQLT PTWRVYSTGS NVFQTRAGCL IGAEHVNNSY ECDIPIGAGI CASYQTQTNS HRRAR
Beta GTNTSNQVAV LYQGVNCTEV PVAIHADQLT PTWRVYSTGS NVFQTRAGCL IGAEHVNNSY ECDIPIGAGI CASYQTQTNS PRRAR
Gamma GTNTSNQVAV LYQGVNCTEV PVAIHADQLT PTWRVYSTGS NVFQTRAGCL IGAEYVNNSY ECDIPIGAGI CASYQTQTNS PRRAR
Delta GTNTSNQVAV LYQGVNCTEV PVAIHADQLT PTWRVYSTGS NVFQTRAGCL IGAEHVNNSY ECDIPIGAGI CASYQTQTNS RRRAR
Epsilon GTNTSNQVAV LYQGVNCTEV PVAIHADQLT PTWRVYSTGS NVFQTRAGCL IGAEHVNNSY ECDIPIGAGI CASYQTQTNS PRRAR
Kappa GTNTSNQVAV LYQGVNCTEV PVAIHADQLT PTWRVYSTGS NVFQTRAGCL IGAEHVNNSY ECDIPIGAGI CASYQTQTNS RRRAR
Iota GTNTSNQVAV LYQGVNCTEV PVAIHADQLT PTWRVYSTGS NVFQTRAGCL IGAEHVNNSY ECDIPIGAGI CASYQTQTNS PRRAR
Eta GTNTSNQVAV LYQGVNCTEV PVAIHADQLT PTWRVYSTGS NVFQTRAGCL IGAEHVNNSY ECDIPIGAGI CASYQTHTNS PRRAR
Lambda GTNTSNQVAV LYQGVNCTEV PVAIHADQLT PTWRVYSTGS NVFQTRAGCL IGAEHVNNSY ECDIPIGAGI CASYQTQTNS PRRAR
Mu GTNTSNQVAV LYQGVNCTEV PVAIHADQLT PTWRVYSTGS NVFQTRAGCL IGAEHVNNSY ECDIPIGAGI CASYQTQTNS HRRAR
Omicron GTNTSNQVAV LYQGVNCTEV PVAIHADQLT PTWRVYSTGS NVFQTRAGCL IGAEYVNNSY ECDIPIGAGI CASYQTQTKS HRRAR

The percentage coverage for each protein determined by Proteome Discoverer is as follows: Alpha 87%; Beta 97%; Gamma 87%; Delta 89%; Epsilon 87%; Kappa 84%; Iota 86%; Eta 70%, Lambda 88%; Mu 85%; and omicron 91%.

*Sections highlighted in yellow show the protein percent coverage
*Amino acid residues in red font are substituted
* "–" Represents deletions
* "↓" Indicates the point of insertion

Supplementary Figure S2

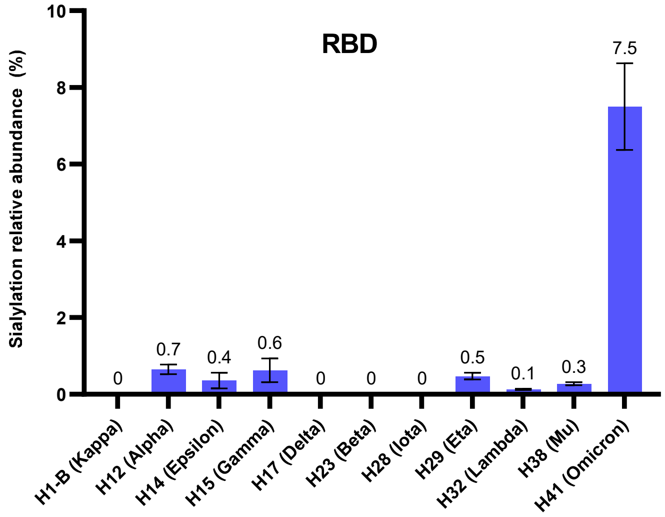

Supplementary Figure S3

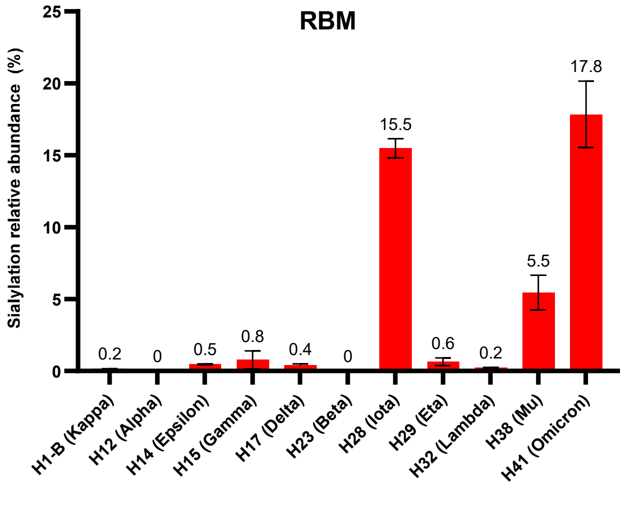

Supplementary Figure S4

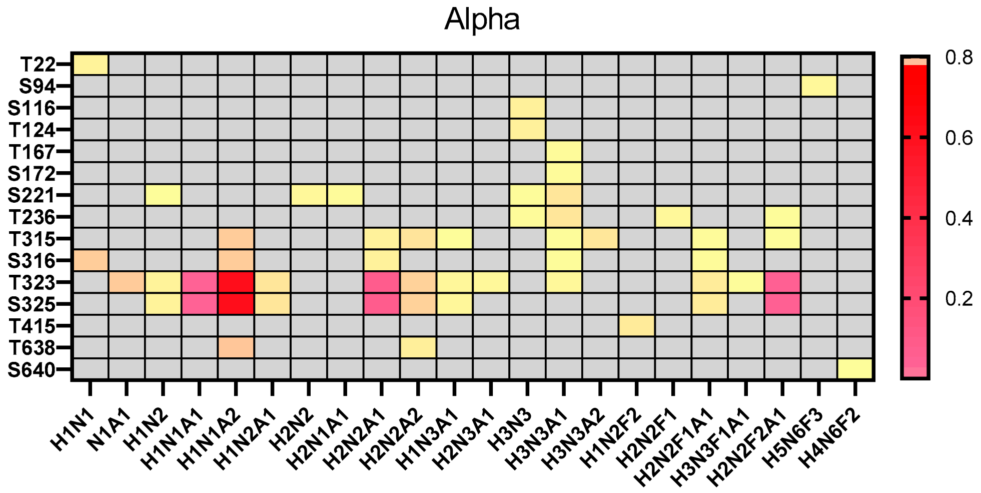

Supplementary Figure S5

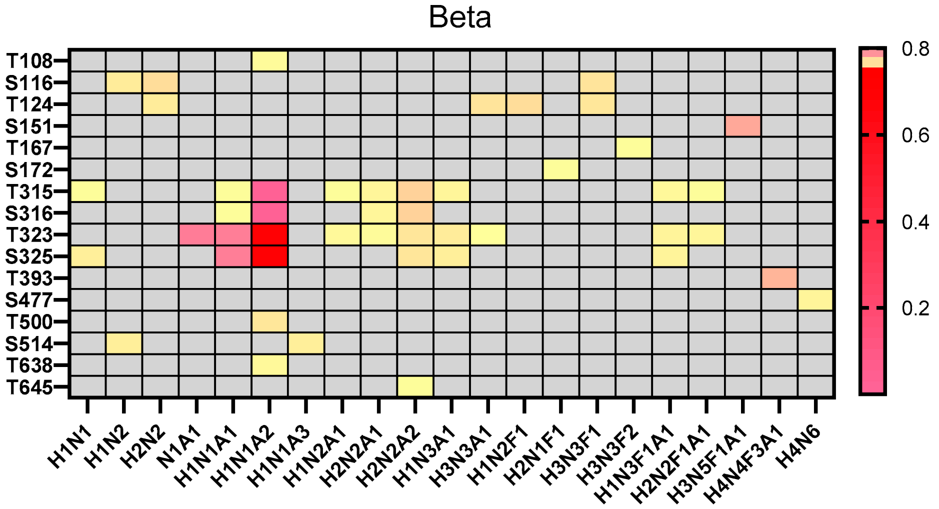

Supplementary Figure S6

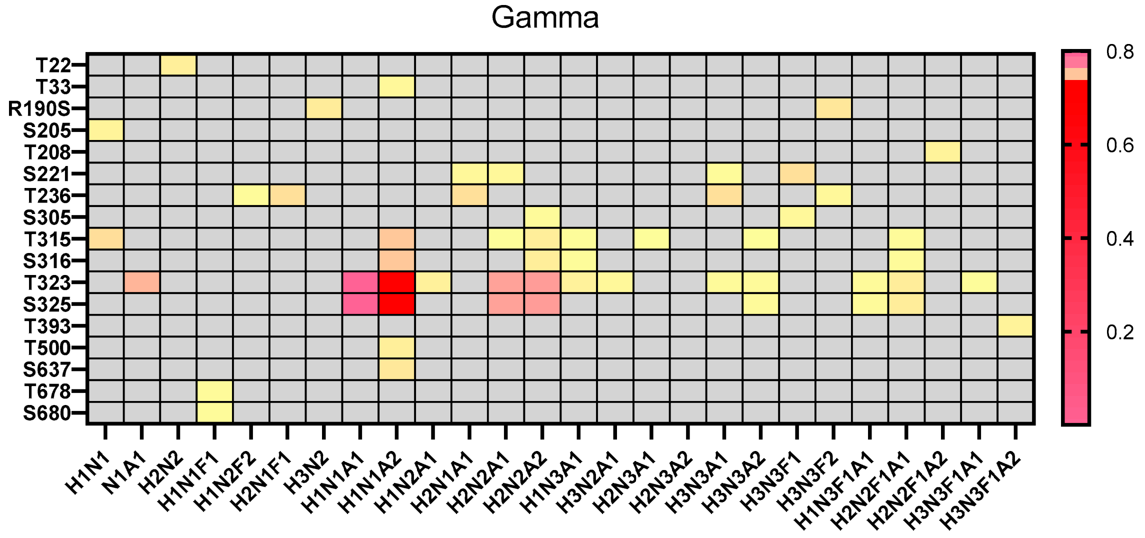

Supplementary Figure S7

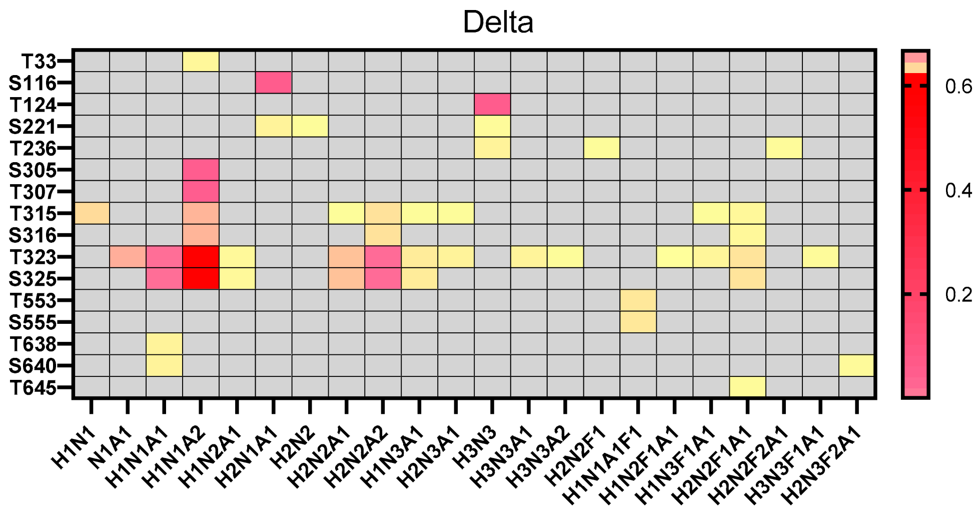

Supplementary Figure S8

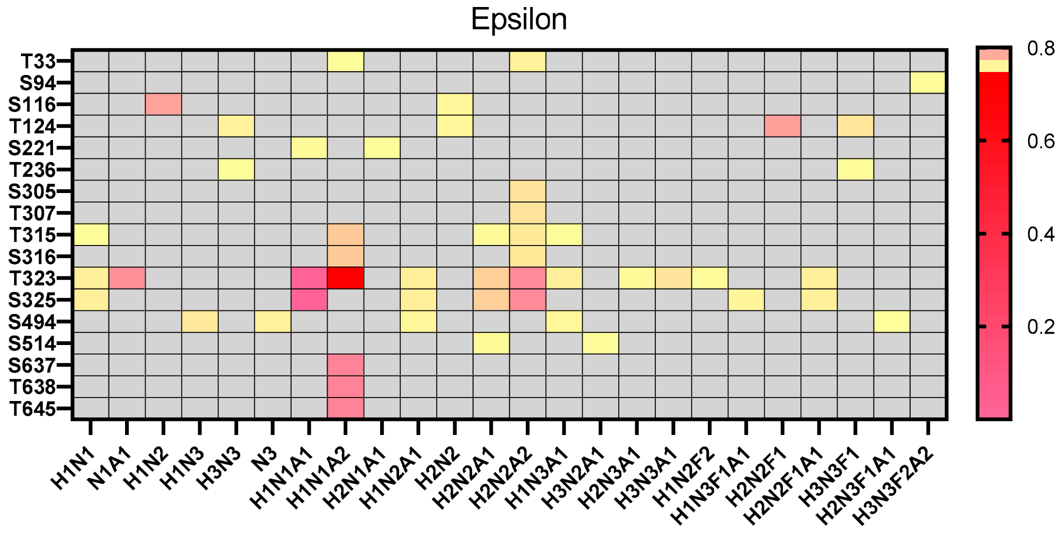

Supplementary Figure S9

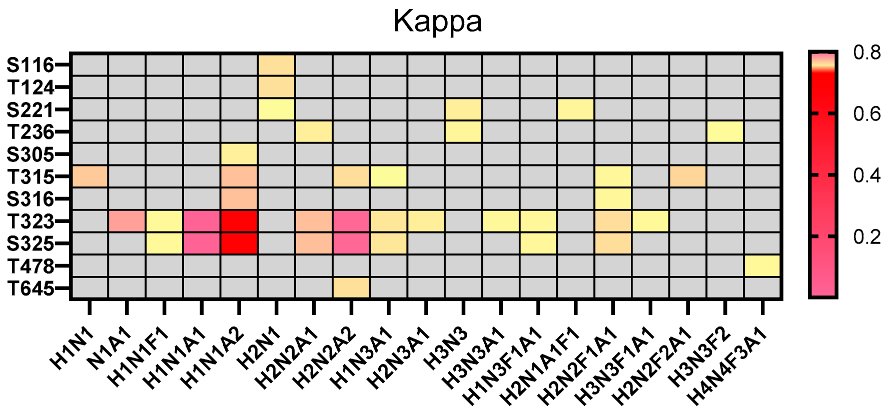

Supplementary Figure S10

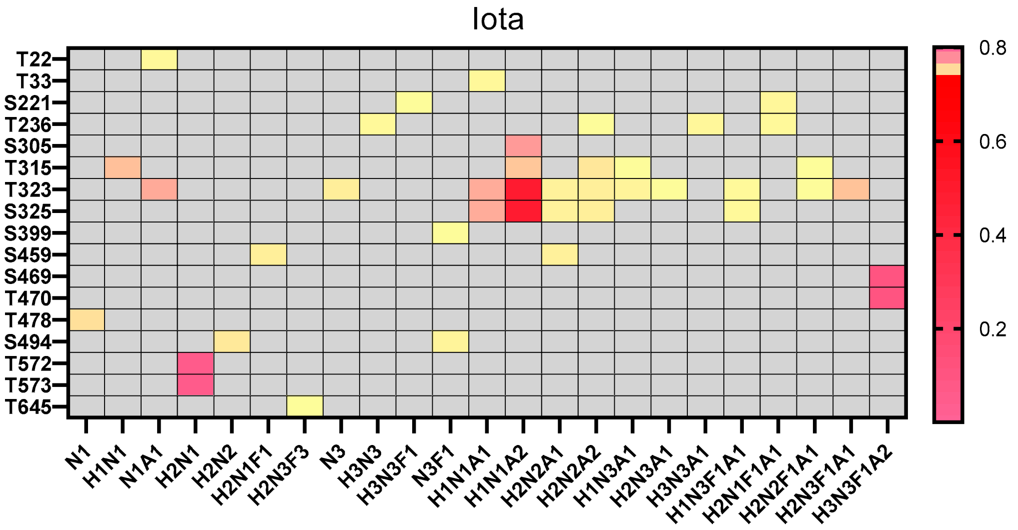

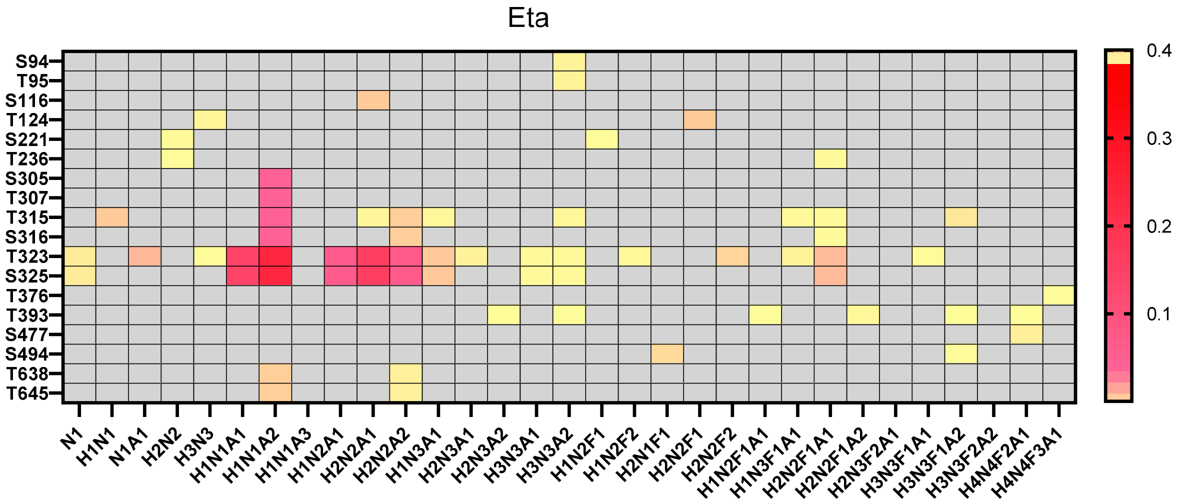
Eta
S94
T95
S116
T124
S221
T236
S305
T307
T315
S316
T323
S325
T376
T393
S477
S494
T638
T645
N1
H1N1
N1A1
H2N2
H3N3
H1N1A1
H1N1A2
H1N1A3
H1N2A1
H2N2A1
H2N2A2
H1N3A1
H2N3A1
H2N3A2
H3N3A1
H3N3A2
H1N2F1
H1N2F2
H2N1F1
H2N2F1
H2N2F2
H1N2F1A1
H1N3F1A1
H2N2F1A1
H2N2F1A2
H2N3F2A1
H3N3F1A1
H3N3F1A2
H3N3F2A2
H4N4F2A1
H4N4F3A1
0.4
0.3
0.2
0.1

Supplementary Figure S12

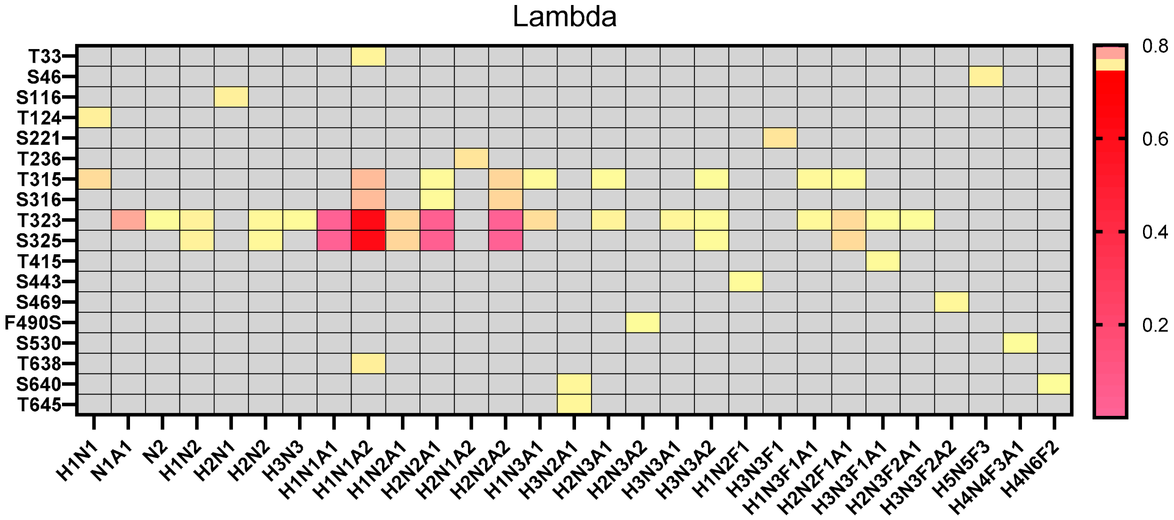

Supplementary Figure S13

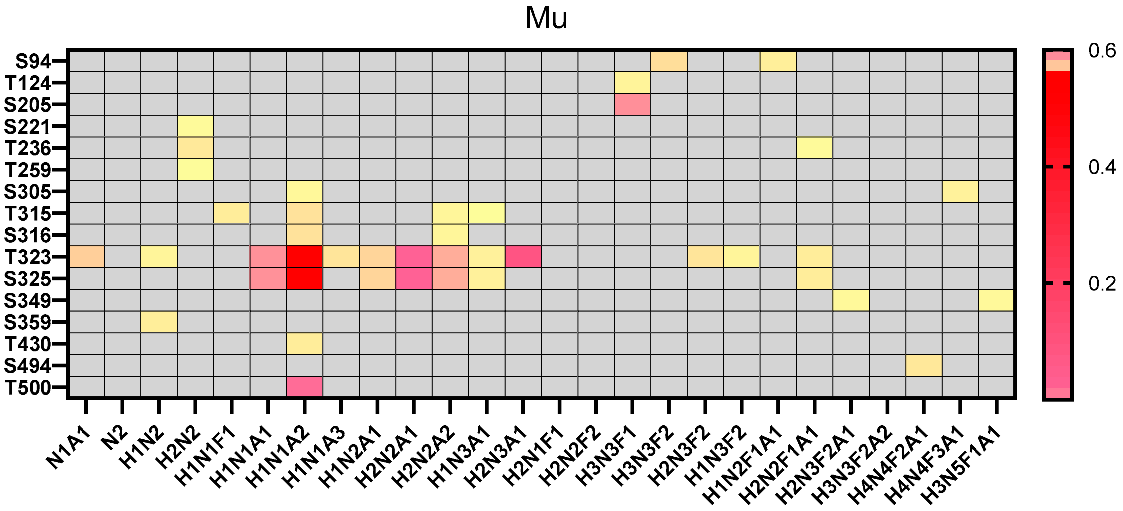

Supplementary Figure S14

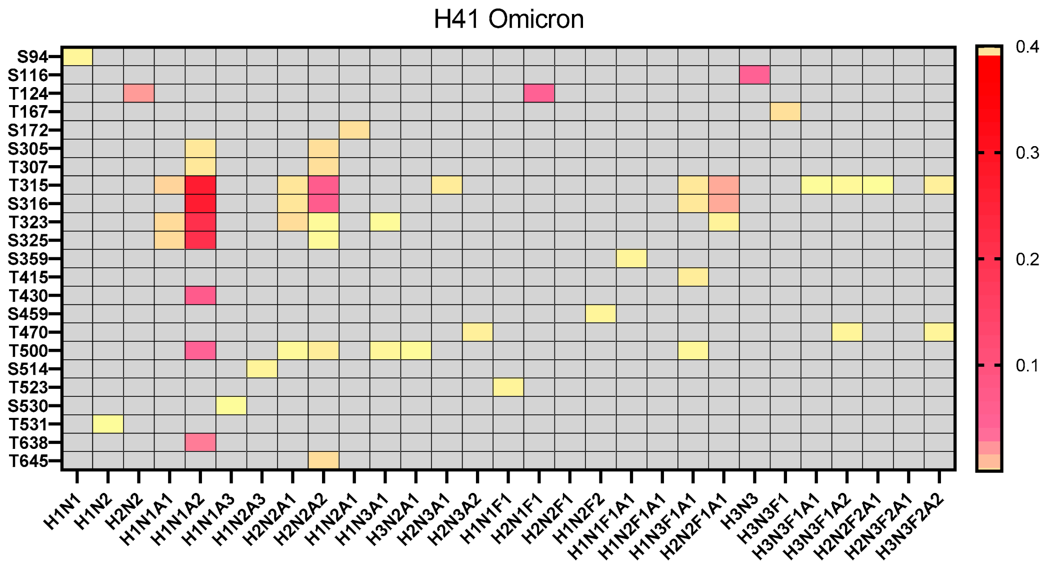

Supplementary Figure S15

(A)

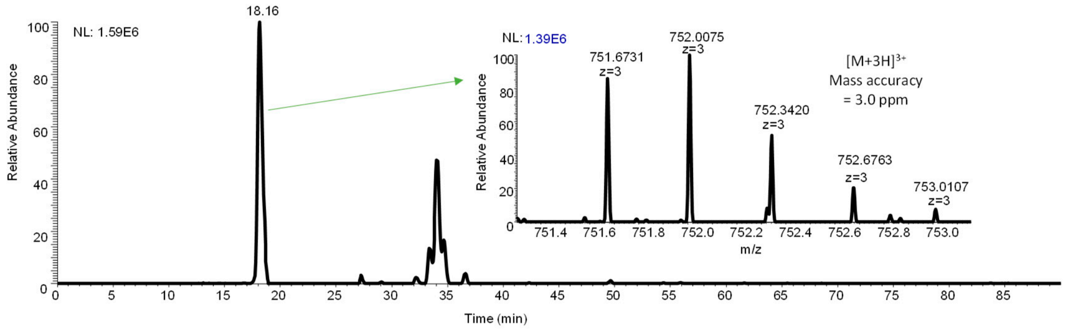

(B)

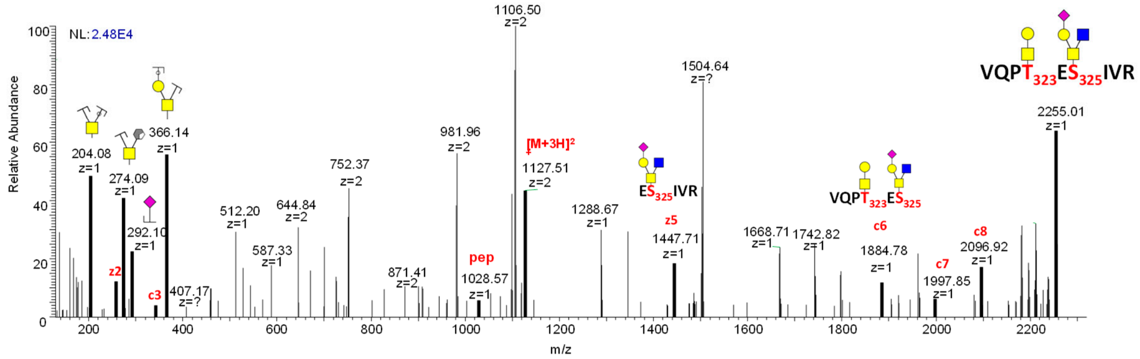

Supplementary Figure S16

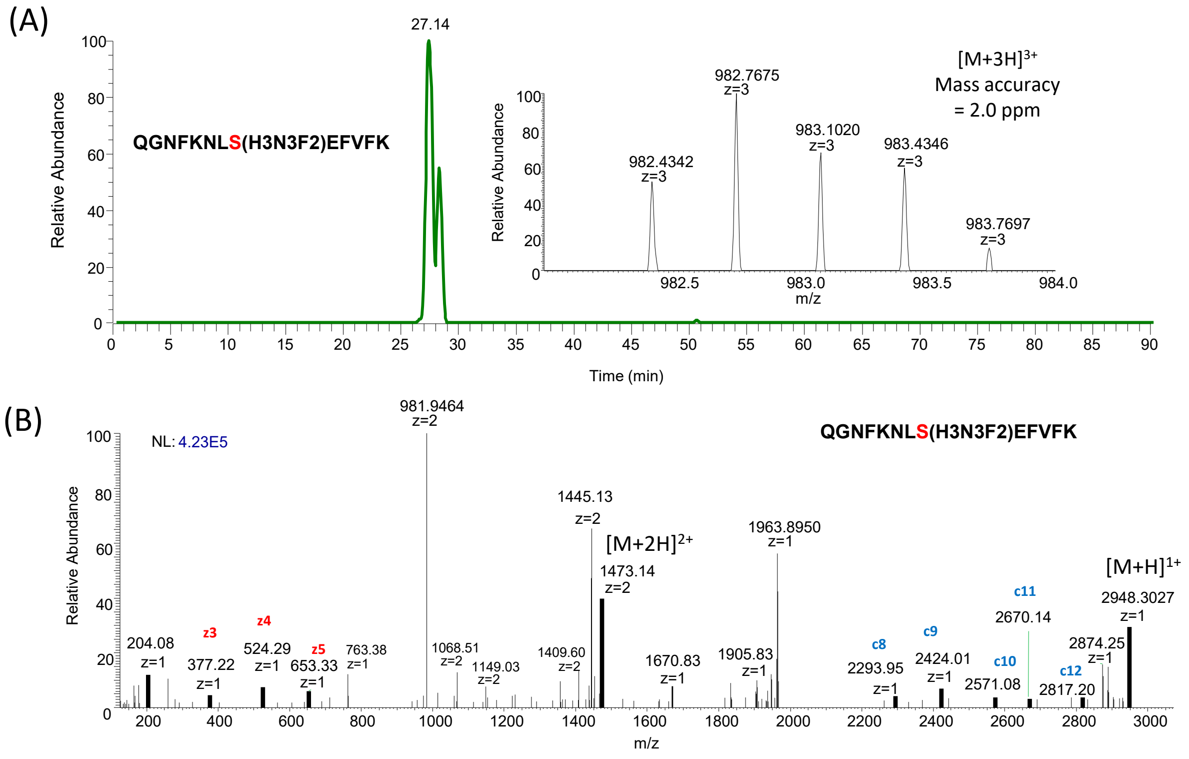

Supplement: Supplementary file 1 [file biomolecules-13-01467-s001.zip › SARS CoV2 O-Glycoproteomics Supplementary Information.pdf]
